# Supplementary material for: Incentive preferences for community health volunteers in Kenya: findings from a discrete choice experiment
Source: BMJ Open. 2021 Jul 5;11(7):e048059. doi: 10.1136/bmjopen-2020-048059 (PMC8258597; doi:10.1136/bmjopen-2020-048059)
Supplement: Author's manuscript [file bmjopen-2020-048059.draft_revisions.pdf]

BMJ Open is committed to open peer review. As part of this commitment we make the peer review history of every article we publish publicly available.

When an article is published we post the peer reviewers' comments and the authors' responses online. We also post the versions of the paper that were used during peer review. These are the versions that the peer review comments apply to.

The versions of the paper that follow are the versions that were submitted during the peer review process. They are not the versions of record or the final published versions. They should not be cited or distributed as the published version of this manuscript.

BMJ Open is an open access journal and the full, final, typeset and author-corrected version of record of the manuscript is available on our site with no access controls, subscription charges or pay-per-view fees (http://bmjopen.bmj.com).

If you have any questions on BMJ Open's open peer review process please email info.bmjopen@bmj.com

BMJ Open

Incentive preferences for community health volunteers in Kenya: Findings from a Discrete Choice Experiment

Journal:	*BMJ Open*
Manuscript ID	bmjopen-2020-048059
Article Type:	Original research
Date Submitted by the Author:	20-Dec-2020

Complete List of Authors:	Abuya, Timothy; Population Council<br>Mwanga, Daniel; Population Council Kenya, Poverty, Gender & Youth<br>Obadha, Melvin; University of Oxford, Nuffield Department of Population Health; KEMRI-Wellcome Trust Research Programme, Nuffield Department of Population Health<br>Ndwiga, Charity ; Population Council<br>Odwe, George; Population Council Kenya<br>Kavoo, Daniel; Minsitry of Health, Kenya, Division of Community Health Services<br>Wanyugu, John; Ministry of Health, Division of Community Health Services<br>Warren, Charlotte; Population Council<br>Agarwal, Smisha; Johns Hopkins Bloomberg School of Public Health, Department of International Health

Keywords:	Health policy < HEALTH SERVICES ADMINISTRATION & MANAGEMENT, International health services < HEALTH SERVICES ADMINISTRATION & MANAGEMENT, Organisation of health services < HEALTH SERVICES ADMINISTRATION & MANAGEMENT

SCHOLARONE™
Manuscripts

I, the Submitting Author has the right to grant and does grant on behalf of all authors of the Work (as defined in the below author licence), an exclusive licence and/or a non-exclusive licence for contributions from authors who are: i) UK Crown employees; ii) where BMJ has agreed a CC-BY licence shall apply, and/or iii) in accordance with the terms applicable for US Federal Government officers or employees acting as part of their official duties; on a worldwide, perpetual, irrevocable, royalty-free basis to BMJ Publishing Group Ltd ("BMJ") its licensees and where the relevant Journal is co-owned by BMJ to the co-owners of the Journal, to publish the Work in this journal and any other BMJ products and to exploit all rights, as set out in our licence.

The Submitting Author accepts and understands that any supply made under these terms is made by BMJ to the Submitting Author unless you are acting as an employee on behalf of your employer or a postgraduate student of an affiliated institution which is paying any applicable article publishing charge ("APC") for Open Access articles. Where the Submitting Author wishes to make the Work available on an Open Access basis (and intends to pay the relevant APC), the terms of reuse of such Open Access shall be governed by a Creative Commons licence – details of these licences and which Creative Commons licence will apply to this Work are set out in our licence referred to above.

Other than as permitted in any relevant BMJ Author's Self Archiving Policies, I confirm this Work has not been accepted for publication elsewhere, is not being considered for publication elsewhere and does not duplicate material already published. I confirm all authors consent to publication of this Work and authorise the granting of this licence.

For peer review only

Incentive preferences for community health volunteers in Kenya: Findings from a Discrete Choice Experiment

Short Title: Understanding community health worker incentive preferences

Timothy Abuya[1], Daniel Mwanga[1], Melvin Obadha[2,3], Charity Ndwiga[1], George Odwe[1], Daniel Kavoo[4], John Wanyugu[4], Charlotte Warren[5] and Smisha Agarwal[6]

1. Population Council, Nairobi, Kenya

2. Health Economics Research Centre, Nuffield Department of Population Health, University of Oxford, Oxford, United Kingdom

3. Health Economics Research Unit, KEMRI-Wellcome Trust Research Programme, Nairobi, Kenya

4 Ministry of Health, Division of Community Health Services, Kenya

5. Population Council, Washington DC

6 Department of International Health, Johns Hopkins Bloomberg School of Public Health, Baltimore, Maryland, USA

Correspondence to:

Dr. Timothy Abuya

Population Council, Kenya

tabuya@popcouncil.org

For peer review only

ABSTRACT

Background

Community Health Volunteers (CHWs) play crucial roles in enabling access to health care at the community levels. Although CHV are considered volunteers, programs provide financial and non-financial incentives but there is limited evidence on which bundle of financial and non-financial incentives are most effective for their improved performance.

Methods

We used discrete choice experiments (DCEs) to understand incentive preferences of CHVs with the aim to improve motivation, performance, and retention of CHVs. Relevant incentive attributes were identified through qualitative interviews with CHVs and with their supervisors. We then deployed a nominal group technique to generate and rank preferred attributes among CHVs. We developed a DCE based on the five attributes administered to 211 CHVs in Kilifi and Bungoma Counties in Kenya. We used conditional logit and mixed multinomial logit models to estimate the utility of each incentive attribute and calculated the trade-offs the CHWs were willing to make for a change in salary.

Results

Transport was the considered the incentive attribute with most relative importance followed by tools of trade then monthly stipend. CHVs preferred job incentives that offered higher monthly stipends even though it was not the most important. They had negative preference for job incentives that provided award mechanisms for the best performing CHVs as compared to jobs that provided recognition at the community level and preferred job incentives that provided more tools of trade compared to those that provided limited tools.

For peer review only

Conclusions

A bundled incentive of both financial and non-financial packages is necessary to provide a conducive working environment for CHVs. The menu of options relevant for CHVs in Kenya include transport, tools of trade and monthly stipend. Policy decisions should be contextualized to include these attributes to facilitate CHW satisfaction and performance.

For peer review only

Strengths and Limitation of the study

Strengths

- DCEs methods enabled us to study incentive packages that did not exist and quantify CHVs preferences for the attributes of incentive alternatives, relative importance they placed on the attributes, and trade-offs of incentive attributes CHVs were willing to make.
- Data generated from this method provide useful information for policy makers on the attributes of a good incentive package for CHVs in Kenya and other similar contexts.
- DCE results illustrated heterogeneity illustrating the value of diversity and how that can be used to ensure context specific adaptation of the menu of incentives that a country can adopt to make use of limited resources

Limitations

- DCEs are prone to bias as they present hypothetical alternatives to respondents. Furthermore, our DCE adopted a forced-choice elicitation format (lack of opt-out) which might have exacerbated hypothetical bias and affected our Willingness to accept estimates.

INTRODUCTION

The shortage of health workers is worsening globally estimated at 18 million health workers by 2030 and 4.3 million in Africa and Asia (1-3). Recruitment and training of community health workers (CHWs) increase access to basic health services including health education where the formal sector falls short (1, 4, 5). Although CHWs roles, recruitment, remuneration and training vary (1), they work within their own community to promote health (6). CHW programs have reported substantial improvements in healthcare outcomes but struggled to maintain quality at scale (5, 7, 8). While CHW are considered volunteers(9), programs provide financial and non-financial incentives (10), facilitating their retention and improved performance (11-13). Integrating CHW into the formal health sector requires adequate incentives (14-16). However, limited evidence exist on which bundle of financial and non-financial incentives are most effective (5, 17).

In Kenya Community Health Services (CHS) are implemented through community health units (CHUs), each serving a population of 5,000 people. Community health volunteers (CHV) who serve these units are chosen by the community and trained to create demand for preventive services. CHV are supervised by a Community Health Extension Workers (CHEW) who provide training and technical support. Currently Kenya has an estimated 6,359 CHUs and 63,590 CHVs. There are 1,500 CHEWs (18). CHS in Kenya increase attendance of antenatal care visits, deliveries by skilled birth attendants, intermittent preventive treatment, testing for HIV during pregnancy, exclusive breastfeeding, and hygiene practices (19, 20). Despite this evidence of effectiveness, CHVs are considered as volunteer health workers. The government directed that they are paid a stipend of KES 2,000 per month (~20 USD), however this compensation continues to be ad-hoc with support from other NGO partners and may occur in the form of transport reimbursement,

For peer review only

lunches or a monthly stipend that is fixed or performance-based. There are efforts to mainstream remuneration of CHVs with a few counties legislating county community health bills that sets aside funding for CHS. Stated preference elicitation methods such as discrete choice experiments (DCEs) can help better understanding of incentive preferences and tradeoffs CHVs are willing to make(21). Financial remuneration as well as non-financial incentives are critical to improve performance and retention of the CHVs, as also recommended by the 2018 WHO guideline on health policy and system support to optimize CHW programs(17). This study uses DCE to understand incentive preferences of CHVs with the aim to improve motivation, performance, and retention of CHVs. Although DCEs have been applied in health research in high-income countries, there are few examples of DCEs used in low income setting for various cadres of health workers (22-27) and especially among CHWs (5, 28, 29). This study provides empirical evidence on the job incentive structure necessary to contribute to the welfare of CHVs using discrete choice experiments (DCE).

METHODS

Phase 1: Identifying attributes and levels

The study was conducted among CHVs from two sub counties of Kilifi, a rural coastal and Bungoma, a western County. Both counties reflect a range of commonly observed barriers to care, including geographical access constraints and cultural vulnerabilities (30). The Counties were purposefully selected in collaboration with County Health Management Teams to include a functional CHS where CHVs conduct routine visits, collect data, and receive some form of incentives. Additionally, the selected sites had several collaborative projects between non-governmental organizations (NGOs) and the government being implemented to strengthen local CHS through capacity building and improve access to health information.

For peer review only - http://bmjopen.bmj.com/site/about/guidelines.xhtml

Phase one identified financial and non-financial attributes and levels that influence CHV performance. We conducted four focus group discussions (FGDs) with CHVs and another four with their supervisors, the CHEWs. FGDs examined their understanding of incentives, preferred incentives, and barriers to implementation and the feasibility of implementing the incentives. We then deployed a nominal group technique to generate and rank preferred attributes among CHVs and CHEWs. At the national level, we conducted four in-depth interviews with policymakers at the division of community health and three key organizations that implement CHS programs.

Validation and refining attributes

Seventeen attributes and their corresponding levels were generated and subjected to a validation process by partners and policy makers at the division of community health. Key considerations used were relevance, ability to compute willingness to accept (WTA) measures, correlations between attributes, importance of an attribute, plausibility, capability of being traded and attribute non-attendance (31, 32). The process resulted in eight attributes which were further reduced to five through a pilot study (table 1).

Table 1: Attributes and attribute levels included in the DCE

**Attribute**	**Attribute level**	**Coding**
Recognition	Recognition at community level (recognition during public meetings)	0 (ref.)
	Recognition at facility (Priority for service provision for them and their families, opportunity to work at facility as volunteer, Acknowledgment of CHV referrals and feedback)	1
	Award mechanism for the best performing CHVs (certification, provision of gifts, wall of fame, opportunities for exchange visit)	2

For peer review only

Income generating activities (IGA)	Provision of seed money/Grant KES 150000/CHU	Continuous
	Provision of seed money/Grant KES 100000/CHU	
Monthly Stipend	KES 2500[1]	Continuous
	KES 4000	
	KES 5500	
Transport	Bicycles for CHV	0 (ref.)
	Motorcycles for CHV per CHU	1
	Motorcycle for CHEW and bicycle for CHV	2
Tools of trade	Supplies and commodities + non-pharmaceutical + job aids/ Information, Education and Communication (IEC) materials	0 (ref.)
	Supplies and commodities + non-pharmaceutical + job aids/IEC materials) + Identification (badges, branded jacket/bag)	**1**
	Supplies and commodities + non-pharmaceutical + job aids/IEC materials) + Identification (badges, branded jacket/bags) + Safety gears (Raincoats, gumboots, and umbrella)	**2**

Experimental design and construction of choice tasks

Phase two comprised a cross sectional quantitative DCE survey among CHVs to elicit their preferences. The choice experiment was unlabeled consisting of two hypothetical incentive alternatives in a forced-choice elicitation format which included full profiles where all five

[1] 1$=Kenya shilling 100

For peer review only

attributes appeared in each alternative. A fractional factorial experimental design was used to generate 12 choice tasks. An orthogonal design was used where each attribute was statistically independent of each other and balanced using Sawtooth Software (33). A check for dominant alternatives was conducted and included as an extra choice task to act as a rationality test and assess internal validity of the data (34). The choice tasks were designed into a survey questionnaire.

DCE survey questionnaire

The survey questionnaire covered background characteristics, sources of income, current incentive structure and compensation, workload, supervision, training, and the choice tasks. The Research Assistants were trained on how to explain each attribute and levels to the participants. They introduced the choice tasks by explaining to the participant the different scenarios and asked given a choice, which of the alternatives they would prefer. This was repeated for each of the choice tasks with participants selecting preferred alternative. The choice tasks were programmed to appear in a random order to minimize social desirability bias (34).

Sampling

The selection of CHVs included in the survey was based on discussions with the sub-county community health focal person considering functionality of each CHU. A rule of thumb by Johnson and Orme (35) was used to determine the minimum sample size represented in the equation below:.

$$N > \frac{500c}{(t * a)}$$

For peer review only

Using a main effects model, a minimum sample size *N* of 84 was derived where *c* (largest number of levels *c* among the five attributes) was four, *t* was 12 choice tasks and *a* was two alternatives. From each ward a maximum of 13-14 active CHVs were selected for the survey. The DCE survey tool was administered to 211 CHVs across Bungoma and Kilifi counties.

Data collection

Data collection was done electronically using Open Data Kit (ODK) Programme by trained interviewers. Before data collection, a field pre-test was conducted to practice the tool and gather experiences on the best approaches of how to explain the choice tasks. The experiences from the field pretest were used by the data collection team to refine how to explain the choice tasks to the CHVs. CHVs were presented with 12 choice tasks prompting them to choose an option they preferred among two incentive alternatives.

Data analysis

Analysis of DCE data follows random utility theory described in the published study protocol (5). A panel mixed multinomial logit model (MMNL) was used to estimate choice probabilities and was preferred over a conditional logit model because it fully relaxes the assumption of independence of irrelevant alternatives (IIA), considers inter respondent preference heterogeneity and within respondent correlation (33, 36). The utility function of the main effects model was specified as follows:

$$U_{njt} = \beta_0 + \beta_1 * Recognition\ at\ facility_{njt} + \beta_2 * Award\ mechanism_{njt} + \beta_3 * Income\ Generating\ Activities_{njt} + \beta_4 * Monthly\ stipend_{njt} + \beta_5 * Motorcycle_{njt} + \beta_6 * Motorcycle\ and\ Bicycle_{njt} + \beta_7 * Identification_{njt} + \beta_8 * Identification\ and\ safety\ gear_{njt} + \varepsilon_{njt}$$

Where U_{njt} was the utility a CHV n derived from selecting incentive alternative j in choice task t, β_0 was the alternative specific constant for alternative A, β_1 to β_8 were parameters to be estimated, ε_{njt} were error terms which were assumed to be IID following type-1 extreme value distribution, IGA and monthly stipend were continuous attributes in Kenya shillings (KES), the rest were dummy coded variables for levels of the recognition, transport, and tools of trade attributes. In the model, all parameters of the attributes were treated as random and normally distributed except IGA and monthly stipend which were restricted to a lognormal distribution. The panel MMNL model resulted in means which represented choice probabilities or preferences and standard deviations which captured preference heterogeneity. The models were stratified by the two counties. Relative importance estimates were computed using the means from the panel MMNL main effects model. We took the absolute value of the mean of each attributes' parameters and multiplied it by the difference between the attribute-levels' highest and lowest values (37). This gave the maximum effect. The relative importance values were then calculated by considering the proportion of the maximum effect in the context of the total for each attribute (37).

To further explore preference heterogeneity, we explored interaction effects in the panel MMNL model by introducing interactions between incentive attributes and CHVs' characteristics; monthly stipend and sex of CHV, monthly stipend and age of CHV, IGA and sex of CHV, and IGA and age of CHV. We computed WTA estimates using a panel MMNL model in willingness to pay (WTP) space (38). We expressed the utility function in WTP space as follows

$$U_{njt} = \beta_{4n}(Monthly\ stipend_{njt} + \beta_0 + \beta_1 * Recognition\ at\ facility_{njt} + \beta_2 * Award\ mechanism_{njt} + \beta_3 * IGA_{njt} + \beta_5 * Motorcycle_{njt} + \beta_6 * Motorcycle\ and\ Bicycle_{njt} + \beta_7 * Identification_{njt} + \beta_8 * Identification\ and\ safety\ gear_{njt}) + \varepsilon_{njt}$$

For peer review only - http://bmjopen.bmj.com/site/about/guidelines.xhtml

The monetary attribute was monthly stipend and represented the amount of money CHVs were willing to accept per month in Kenya shillings (KES). WTA estimates were stratified by county. All models used 1000 Halton draws. Analysis was conducted using Mixlogit and Mixlogitwtp commands on Stata 15.1(39). Chi-square was used to compare the categories for the descriptive statistics.

Ethical Approval

The research protocol was approved by the Population Council's Institutional Review Board (PC IRB 872) and AMREF Ethical Review Board ESRC P572-2018. All the participants gave written consent for the DCE survey.

Patient and Public Involvement

There was no patient involvement

RESULTS

Characteristics of respondents

Of 211 CHVs interviewed, the majority were female (70%), aged 46 or more years, and had secondary education (62%). More than a half (56%) reported aspiring to do business and about a third wanted to continue working as CHVs. The majority (91%) reported having been engaged in other economic activities, before becoming CHV, mainly doing small scale business and agricultural (Table 2).

Table 2: Characteristics of CHV interviewed

	Kilifi		Bungoma		Total		p-value
**Gender**	**n=109**	**%**	**n=102**	**%**	**n=211**	**%**	
Male	38	34.9	26	25.5	64	30.3	
Female	71	65.1	76	74.5	147	69.7	0.139
**Age (years)**							
27-35	22	20.2	18	17.6	40	19	
36-45	36	33	32	31.4	68	32.2	0.813

For peer review only - http://bmjopen.bmj.com/site/about/guidelines.xhtml

46 or above	51	46.8	52	51	103	48.8	
**Highest Education Level**							
Primary	52	47.7	9	8.8	61	28.9	
Secondary	47	43.1	84	82.4	131	62.1	
College/University/Vocational	10	9.2	9	8.8	19	9	<0.001
**Future Career plans**							
Starting a business	68	62.4	49	48	117	55.5	0.036
Continue working as CHV	47	43.1	21	20.6	68	32.2	<0.001
Other options	25	22.9	28	27.5	53	25.1	0.45
Health related work	17	15.6	21	20.6	38	18	0.346
Further Study	10	9.2	6	5.9	16	7.6	0.367
Becoming a political Leader	2	1.8	1	1	3	1.4	0.6
**Marital Status**							
Married/living together	81	74.3	93	91.2	174	82.5	
Widowed	12	11	6	5.9	18	8.5	
Never married	6	5.5	2	2	8	3.8	
Divorced/separated	10	9.2	1	1	11	5.2	0.007
**Working before becoming CHV (%)**	92	84.4	99	97.1	191	90.5	0.002
**Activities engaged before becoming CHV**							
Business owner	49	53.3	41	41.4	90	47.1	
Agriculture	12	13	63	63.6	75	39.3	
Other	32	34.8	15	15.2	47	24.6	
Community/Religious Leader	5	5.4	4	4	9	4.7	<0.001
Teaching	2	2.2	5	5.1	7	3.7	
Other health professional	1	1.1	5	5.1	6	3.1	
Village Savings Group	1	1.1	0	0	1	0.5	
**Time taken to farthest household**							
Less than 15 minutes	11	10.1	4	3.9	15	7.1	
15-less than 30 minutes	44	40.4	27	26.5	71	33.6	
30-less than 60 minutes	38	34.9	52	51	90	42.7	
1 hour -less than 2 hours	15	13.8	11	10.8	26	12.3	
2 hours or more	1	0.9	8	7.8	9	4.3	0.004

Current incentive structure for CHVs in Kenya

Table 3 shows that nearly all the CHVs interviewed (98%) receive some form of compensation either financial or non-financial which are often infrequent (44.2%). Among those reporting receipt of financial compensation, less than 15% received amounts that were within the

For peer review only

recommended range of KES 2000 (US $ 20) per month while about 40% receive about KES 5000 (US $ 50) per month.

Table 3: Type of incentives currently provided

	Kilifi		Bungoma		Total		p-value
**% receiving**	**n=109**	**%**	**n=102**	**%**	**n=211**	**%**	
Any form of compensation	107	98.2	100	98	207	98.1	0.947
**Type of compensation received**							
Financial	71	66.4	64	64	135	65.2	
Non-financial	1	0.9	0	0	1	0.5	
Both	35	32.7	36	36	71	34.3	0.565
**Frequency of receipt of compensation**							
Monthly	23	21.7	11	11	34	16.5	
Quarterly	0	0	79	79	79	38.3	
Semi-Annual	2	1.9	0	0	2	1	
Ad hoc	81	76.4	10	10	91	44.2	<0.001
**Amount received per month**							
< KES1000	86	81.1	3	3	89	43.2	
KES 1000-2499	12	11.3	17	17	29	14.1	
KES 2500 - 4999	5	4.7	1	1	6	2.9	
KES 5000 or more	3	2.8	79	79	82	39.8	<0.001
**Sources of Financial Compensation**							
Government	9	8.5	91	91	100	48.5	<0.001
NGO	99	93.4	19	19	118	57.3	<0.001
Community members	0	0.0	0	0.0	0	0.0	NA
Do not know	2	1.9	0	0	2	1	NA
**Type of Non-Financial Compensation**							
Food, clothes /other material goods	33	91.7	10	27.8	43	59.7	<0.001
Supplies for work	7	19.4	23	63.9	30	41.7	<0.001
Additional training	2	5.6	12	33.3	14	19.4	0.003
Other forms	3	8.3	2	5.6	5	6.9	0.643
Health services	0	0	1	2.8	1	1.4	NA
**Sources of Non-Financial Compensation**							
Government	10	27.8	3	8.3	13	18.1	0.032
NGO	29	80.6	31	86.1	60	83.3	0.527
Community members	1	2.8	4	11.1	5	6.9	0.164

Notes: US $ 1 = KES 100

CHV preferences and relative importance estimates

For peer review only

Table 4 provides the preference estimates of the incentives attributes. Overall, as well in independently in Kilifi and Bungoma counties, CHVs had a negative preference for jobs that provided motorcycles per CHU compared to those that provided bicycles only. Finally, CHVs significantly preferred jobs that provided more tools of trade to those that provided limited tools. Overall, there was significant heterogeneity in preference across respondents for most attributes as denoted by the statistically significant standard deviation.

For peer review only

Table 4: Main effects panel Mixed Multinomial Logit Model: Preference estimates

**Attribute**	**Attribute Level**	**Total Sample (n=211)**		**Kilifi (n=109)**		**Bungoma (n=102)**	
ASC		β	SE	β	SE	β	SE
	ASC μ	0.127	0.196	-0.204	0.274	0.601	0.319
	ASC σ	-0.049	0.098	0.013	0.111	0.128	0.391
**Recognition**	**Recognition at community level (Ref)**						
	Recognition at facility μ	-0.056	0.178	0.374	0.265	-0.532	0.285
	Recognition at facility σ	1.453**	0.264	1.574**	0.402	1.547**	0.449
	Award Mechanism for best performing CHV μ	-0.670**	0.193	-0.582*	0.266	- 0.901**	0.333
	Award Mechanism for best performing CHV σ	1.541**	0.212	1.718**	0.328	1.592**	0.361
Transport	**Bicycles for CHV (Ref)**						
	Motorcycle for CHV/CU μ	-2.439**	0.247	- 2.368**	0.364	- 2.825**	0.407
	Motorcycle for CHV/CU σ	2.103**	0.272	2.427**	0.407	1.814**	0.467
	Motorcycle for CHEW and bicycle for CHV μ	0.285	0.314	-0.552	0.431	1.466**	0.540
	Motorcycle for CHEW and bicycle for CHV σ	0.464	0.363	0.121	0.276	- 1.061**	0.412
Tools of trade	**Supplies and commodities+ non-pharmaceutical + job aids/IEC materials) (Ref)**						
	Supplies and commodities+ non-pharmaceutical + job aids/IEC materials) + Identification μ	1.385**	0.165	1.466**	0.238	1.376**	0.253
	Supplies and commodities+ non-pharmaceutical + job	-0.669**	0.222	-0.183	0.210	1.029*	0.410

	aids/IEC materials) + Identification σ						
	Supplies and commodities+ non-pharmaceutical + job aids/IEC materials) + Identification + Safety gears μ	2.311**	0.230	2.849**	0.375	1.901**	0.357
	Supplies and commodities+ non-pharmaceutical + job aids/IEC materials) + Identification + Safety gears σ	1.150**	0.239	1.203**	0.321	1.214**	0.428
Monthly Stipend	Monthly stipend μ	0.543**	0.090	0.445**	0.111	0.791*	0.156
	Monthly stipend σ	0.794**	0.242	0.843	0.576	0.960**	0.316
Income Generating Activities	IGA μ	-0.233	0.213	-0.258	0.278	-0.314	0.284
	IGA σ	0.060	0.129	0.502	0.773	0.191	0.175
**Model Fit**							
**# observations**		**5064**		**2,616**		**2,448**	
Log Likelihood		-1087.92		-553.42		-520.56	
Wald Chi2		252.91		97.31		142.70	
Pseudo R^2		0.31					
Prob > chi2		<0.001		<0.001		<0.001	
*Values of monthly stipend are in thousands while IGA are in hundred thousand. Statistical significance: ** at 0.01 level, * at 0.05 level*							

*Note: Across all tables unless stated otherwise: β – Coefficient, # denotes number, ASC- Alternative Specific Constant, Statistical significance: ** at 0.01 level, * at 0.05 level. μ is mean and σ is standard deviation. All attributes were random and normally distributed except monthly stipend and IGA which were restricted to a lognormal distribution. All models used 1000 Halton draws*

Relative importance estimates indicate that transport was considered the most important incentive attribute followed by tools of trade and monthly stipend. The least important incentive attribute was IGA. However, in Kilifi County, CHV considered tools of trade as the most important attribute followed by transport and monthly stipend. This was slightly different from Bungoma County who CHVs rated transport as the most important attribute followed by monthly stipend and then tools of trade.

For peer review only

CHVs WTA estimates

Considering WTA estimates, table 5 shows that in the whole sample, CHVs were significantly willing to accept a KES 1030 (US $ 10.30) increase in monthly stipend for an incentive package that awarded the best performing CHV to one that provided recognition at the community level if everything else was kept constant. Additionally, CHVs were willing to accept a KES 4811 (US $ 48.11) increase in monthly stipend for an incentive package that provided motorcycles to one that provided bicycles only. The magnitude of the increase was greater among CHVs in Kilifi county KES 5834 (US $ 58.34) than Bungoma KES 3913 (US $ 39.13).

Table 5: Main effects panel Mixed Multinomial Logit Model in WTP space

		**Total Sample (n=211)**		**Kilifi (n=109)**		**Bungoma (n=102)**	
Attribute	Attribute Level	β	SE	β	SE	β	SE
ASC	ASC μ	0.011	0.406	0.929	0.837	-0.462	0.253
	ASC σ	-0.156	0.372	-0.820	0.591	-0.355	0.197
Recognition	**Recognition at community level (Ref)**						
	Recognition at facility μ	0.011	0.364	-1.092	0.597	0.745	0.409
	Recognition at facility σ	-2.652**	0.856	3.602**	1.314	-2.249**	0.355
	Award Mechanism for best performing CHV μ	1.030*	0.455	0.708	0.63	0.769*	0.343
	Award Mechanism for best performing CHV σ	-3.005**	0.707	4.108**	1.315	-1.815**	0.181
Transport	**Bicycles for CHV (Ref)**						
	Motorcycle for CHV/CU μ	4.811**	0.799	5.834**	1.406	3.913**	0.339
	Motorcycle for CHV/CU σ	4.232**	0.811	6.018**	1.644	2.523**	0.336
	Motorcycle for CHEW and bicycle for CHV μ	-0.141	0.649	1.982	1.425	-1.318**	0.378

	Motorcycle for CHEW and bicycle for CHV σ	-0.819	0.638	0.507	1.867	1.188**	0.185
Tools of trade	**Supplies and commodities+ non-pharmaceutical + job aids/IEC materials) (Ref)**						
	Supplies and commodities+ non-pharmaceutical + job aids/IEC materials) + Identification μ	-2.610**	0.640	-3.283**	1.257	-1.502**	0.311
	Supplies and commodities+ non-pharmaceutical + job aids/IEC materials) + Identification σ	-0.111	0.374	-0.284	0.279	-0.563*	0.276
	Supplies and commodities+ non-pharmaceutical + job aids/IEC materials) + Identification + Safety gears μ	-4.433	1.082	-6.426**	1.983	-2.172**	0.345
	Supplies and commodities+ non-pharmaceutical + job aids/IEC materials) + Identification + Safety gears σ	2.011**	0.725	-2.366*	1.108	1.297**	0.207
Monthly Stipend	Salary μ	0.577**	0.143	0.564*	0.242	2.001	1.339
	Salary σ	0.308**	0.155	0.351*	0.304	2.915**	3.661
IGA	IGA μ	0.486	0.396	0.796	0.481	0.434	0.304
	IGA σ	-0.400	0.170	-1.523**	0.311	-0.049	0.306
Model Fit							
# observations		5064		2616		2,448	
Log Likelihood		-1100.46		-557.30		-525.17	
Wald Chi2		79.09		79.09		341.71	
Pseudo R^2							
Prob > chi2		<0.001		<0.001		<0.001	

The monetary attribute was monthly stipend and the WTA values are in thousands of Kenya Shillings (KES). US$ 1 = KES 100
*Statistical significance: ** at 0.01 level, * at 0.05 level*

Furthermore, CHVs in the whole sample were willing to accept a KES 2610 (US $ 26.10) reduction in monthly stipend for an incentive package that additionally provided Identification and a KES 4433 (US $ 44.33) decrease for an incentive package that additionally provided identification and safety gears to one that only provided supplies and commodities, non-pharmaceuticals, and job aids when everything else was kept constant. These reductions in monthly stipends were also greater in Kilifi County than Bungoma. Overall, there was significant heterogeneity in WTA estimates across respondents in most attributes as denoted by the statistically significant standard deviations.

Interactions

Table 6 shows the model that present results of the interactions between incentive attributes and CHVs' characteristics. Results show that younger CHVs significantly preferred higher salaries when everything else was kept constant. The interaction between monthly stipend and gender suggested that male CHVs preferred higher salaries than their female counterparts if everything else is kept constant. However, this interaction was not statistically significant. Overall, both interactions revealed significant heterogeneity in preferences across respondents.

For peer review only

Table 6: Panel Mixed Multinomial Logit Model with Interactions (Sex and Age of the CHV): Preference estimates

Attribute	Attribute Level	β	SE
ASC μ		0.119	0.202
ASC σ		0.001	0.217
Recognition	Recognition at community level (Ref)		

	Recognition at facility μ	-0.124	0.187
	Recognition at facility σ	1.611**	0.307
	Award Mechanism for best performing CHV μ	-0.763**	0.208
	Award Mechanism for best performing CHV σ	1.654**	0.273
Transport	**Bicycles for CHV (Ref)**		
	Motorcycle for CHV/CU μ	-2.576**	0.302
	Motorcycle for CHV/CU σ	2.233**	0.308
	Motorcycle for CHEW and bicycle for CHV μ	0.300	0.324
	Motorcycle for CHEW and bicycle for CHV σ	-0.473	0.531
Tools of trade	**Supplies and commodities+ non-pharmaceutical + job aids/IEC materials) (Ref)**		
	Supplies and commodities+ non-pharmaceutical + job aids/IEC materials) + Identification μ	1.418**	0.174
	Supplies and commodities+ non-pharmaceutical + job aids/IEC materials) + Identification σ	0.229	0.455
	Supplies and commodities+ non-pharmaceutical + job aids/IEC materials) + Identification + Safety gears μ	2.375**	0.263
	Supplies and commodities+ non-pharmaceutical + job aids/IEC materials) + Identification + Safety gears σ	1.086**	0.248
Monthly Stipend	Monthly stipend μ	0.964**	0.235
	Monthly stipend σ	0.540**	0.096
IGA	IGA μ	-1.107	0.844
	IGA σ	0.252	0.180
**Interactions**			
	Monthly stipend ##Female μ	-0.151	0.100
	Monthly stipend ##Female σ	0.180**	0.049
	IGA ##Female μ	0.180	0.320
	IGA ##Female σ	0.117	0.145
	Monthly stipend ##Age μ	-0.007**	0.004
	Monthly stipend ##Age σ	0.005**	0.001
	IGA ##Age μ	0.016	0.016

	IGA ##Age σ	0.005	0.006
**Model Fit**			
# observations		5064	
Log Likelihood		-1083.38	
Wald Chi2		801.78	
Pseudo R^2			
Prob > chi2		<0.001	
**Notes:** ##-Interactions; Age is in years.<br>*Statistical significance: ** at 0.01 level, * at 0.05 level*			

For peer review only

Discussion

Our study shows that transport was considered the most important attribute, followed by tools of trade and monthly stipend. Some variations in these preferences were observed between Kilifi and Bungoma counties perhaps due to rurality and varying social-economic contexts. Critical variations in preferences were observed based on CHV's age and gender. Our result shows that older CHVs significantly preferred incentives with lower salaries when everything else was kept constant. Perhaps because older CHV may have experienced voluntariness for long and are therefore content with lower salaries. Younger male CHVs had a stronger preference for higher salaries, compared to older female CHVs, an observation associated with gender roles, perceptions of workload, resources, and logistics, which are barriers to CHW performance (40). It is plausible that gender roles affect salary preference and may have implications on retention, a policy issue that any incentive structure need to consider (41). Workload on the other hand was discussed in the context of use of paper tools or CHWs covering large areas with expectations of reporting household visits monthly regardless of spread, limiting their ability to engage in personal activities and responsibilities (42). These result contrasts with studies from India which showed that age did not affect CHW salary preferences(43). Overall, heterogeneity in preferences across

respondents is a feature that has been reported elsewhere with preferences being influenced by education level, having another paid job or being the main household earner(43).

CHVs had a negative preference for incentive that provided recognition at the facility and award mechanisms for the best performing CHVs as compared to jobs that provided recognition at the community level. These results are augmented by WTA estimates, which suggest that CHVs were willing to accept a KES 1030 (US $ 10.30) increase in monthly stipend for a job that awarded the best performing CHV to one that provided recognition at the community level if everything else was kept constant. Recognition was discussed as a key motivator that will enhance their work. For example, recognition at community level could be as simple as being given a chance to speak at the community public events or benchmarking to other sites to share learnings. Since CHWs are often believed to be vehicles for facilitating community agency and triggering social change(44), recognition is central to maintaining trust between communities and CHW. Community recognition has been shown to influence their status in their locality which might confer other benefits beyond their role as CHVs as has been reported in Uganda with an enhanced status being conferred to them that derive pride and greater access to help or information and being consulted on a range of problems(29). However, we observed variations in role of recognition with geographical location. For example, Kilifi County, a coastal mixed urban and rural settings unlike Bungoma an agrarian setting preferring jobs that provided recognition at the facility to recognition at community. These differences might be due to cultural orientation or benefits accrued from serving in the facility that might elevate CHVs to a same status as health facility-based providers.

For peer review only

Lack of transport for CHWs is reported as a factor limiting their work performance (29, 40). Our results show that CHVs had a negative preference for jobs that provided motorcycles to be used by the CHU and positive preference for jobs that provided motorcycles to the CHU and bicycles to the CHV as compared to those that provided bicycles only. These results illustrate the value of transport that facilitates CHV to conduct routine visits. Preferences for bicycles for themselves and a motorcycle per CHU is probably based on the ability to improve community-facility linkages by transportation of clients to the hospital. Community referrals to a facility is a major barrier to optimal service use where clients who do not have means of transport fail to comply with referrals leading to poor outcomes. Motorcycles can also be used communally as a source of income when not busy serving patients. However, issues of maintenance cost and managing communally owned motorbikes may be an avenue for disagreement and might be a complex policy option to implement. These results echo experiences from Uganda which showed the CHW prefer programs with transport in form of bicycles, which would facilitate visiting clients or going to the health center for supervisory meetings or access supplies but if nonexistent conferred additional financial cost(29).

CHVs significantly preferred jobs that provided more tools of trade to those that provided limited tools with significant heterogeneity in preference across respondents. Having reporting tools and job aids replenished timely facilitate accuracy in reporting as well, orientation to new updates and facilitate CHW performance. Provision of identification materials such as T-shirt, Badges, Caps, branded bags, reflector jackets were described as useful especially during the rainy seasons to maintain the training materials. In addition, provision of essential requirements in the form of CHVs Kit including pharmaceuticals and non-pharmaceuticals for work were considered important. This may mean that policies that provide CHV with essential tools of trade is likely to

For peer review only

influence their performance but will have to be structured in a way that ensures limited stock outs to facilitate continuity of service to communities. In summary other studies have shown that transport constraints and lack of supplies hinder CHW performance(29, 45), thus a useful non-financial incentive.

Finally, relative importance estimates indicate that transport was considered the most important attribute followed by tools of trade and monthly stipend. The least important attribute was IGA. These findings have significant policy direction in the sense that an incentive structure which combines effective transport system for CHV, essential tools of trade to enable them to perform their roles and monthly stipend will be critical for the success of CHV program. However, the levels may need to be contextualized to ensure that the implementation is feasible and sustainable. It might also be important to consider geographical variations even within a country. For example, relative importance varied with Kilifi County considered tools of trade as the most important attribute followed by transport and monthly stipend while Bungoma CHVs rated transport as the most important attribute followed by monthly stipend and then tools of trade. These findings support the growing evidence of the importance of the non-financial interventions in motivating CHWs in developing countries(43).

DCEs are a stated preference approach that enabled us to study incentive packages that did not exist. We were able to quantify CHVs preferences for the attributes of incentive alternatives, relative importance they placed on the attributes, and quantify trade-offs of incentive attributes CHVs were willing to make. This provides useful information for policy makers on the attributes of a good incentive package for CHVs in Kenya and other similar contexts. However, since DCEs are stated preference approach, they are prone to bias as they present hypothetical alternatives to

For peer review only

respondents. Furthermore, our DCE adopted a forced-choice elicitation format (lack of opt-out) which might have exacerbated hypothetical bias and affected our WTA estimates(46). Nonetheless, we used qualitative methods to identify and validate our attributes and levels with potential study participants and policy makers which might have reduced hypothetical bias. A potential value of the DCE beyond the menu of incentives options that CHW prefer may be to practically test the results by implementing the preferred incentive attributes and assessing how it influences the motivation and their retention. Although our results show heterogeneity in terms of incentive preferences for CHVs in different locations, the strength is that it illustrates the value of diverse settings and how that can be used to ensure context specific adaptation of the menu of incentives that a country can adopt to make use of limited resources.

Conclusion

Our study confirms that a bundled incentive of both financial and non-financial packages is necessary to provide a conducive working environment for CHVs. The menu of options relevant for CHVs in Kenya include transport that was considered the most important attribute followed by tools of trade and monthly stipend. The least important attribute was IGA. Policy decisions should be contextualized to include these attributes to facilitate CHW satisfaction and performance.

For peer review only

Authorship contributions

TA co-conceptualized the study, led the analysis, and drafted the manuscript. DM Supported in data collection, overall study implementation, data management analysis and reviewed the manuscript. MO supported in technical design of the DCE experiment, guided analysis and reviewed the manuscripts for technical content. CN supported in design of the DCE experiment and reviewed manuscript. GO supported in technical design of the DCE experiment and reviewed the manuscripts for technical content. DK and JW supported design of the DCE experiment and reviewed the manuscripts for technical content. CW and SA are lead investigators in the overall study, co-designed the DCE study, framing of this manuscript, and contributed significantly to writing and reviewing drafts. All contributing authors provided inputs and approved the final draft for submission.

Competing interests

Authors have no competing interests

Funding

This paper is based on research funded by the Bill & Melinda Gates Foundation. The findings and conclusions contained within are those of the authors and do not necessarily reflect positions or policies of the Bill & Melinda Gates Foundation

Data sharing statement

All the data contributing to this manuscript have been archived in accordance with Population Council data sharing policies. Data will be made available upon reasonable request from the lead Author.

For peer review only

Reference

1. O'Donovan J, O'Donovan C, Kuhn I, Sachs SE, Winters N. Ongoing training of community health workers in low-income andmiddle-income countries: a systematic scoping review of the literature. BMJ Open. 2018;8(4):e021467.
2. Darzi A, Evans T. The global shortage of health workers-an opportunity to transform care. Lancet. 2016;388(10060):2576-7.
3. Global strategy on human resources for health: Workforce 2030 [press release]. 2016.
4. Perry HB, Zulliger R, Rogers MM. Community health workers in low-, middle-, and high-income countries: an overview of their history, recent evolution, and current effectiveness. Annu Rev Public Health. 2014;35:399-421.
5. Agarwal S, Anaba U, Abuya T, Kintu R, Casseus A, Hossain S, et al. Understanding incentive preferences of community health workers using discrete choice experiments: a multicountry protocol for Kenya, Uganda, Bangladesh and Haiti. BMJ Open. 2019;9(12):e033601.
6. Turinawe EB, Rwemisisi JT, Musinguzi LK, de Groot M, Muhangi D, de Vries DH, et al. Selection and performance of village health teams (VHTs) in Uganda: lessons from the natural helper model of health promotion. Hum Resour Health. 2015;13:73.
7. Bang AT, Bang RA, Baitule SB, Reddy MH, Deshmukh MD. Effect of home-based neonatal care and management of sepsis on neonatal mortality: field trial in rural India. Lancet. 1999;354(9194):1955-61.
8. Chowdhury AM, Chowdhury S, Islam MN, Islam A, Vaughan JP. Control of tuberculosis by community health workers in Bangladesh. Lancet. 1997;350(9072):169-72.
9. Maes K. Examining health-care volunteerism in a food- and financially-insecure world. Bull World Health Organ. 2010;88(11):867-9.
10. Hongoro C, McPake B. How to bridge the gap in human resources for health. Lancet. 2004;364(9443):1451-6.
11. Nsabagasani X, Jesca Nsungwa S, Kallander K, Peterson S, Pariyo G, Tomson G. Home-based management of fever in rural Uganda: community perceptions and provider opinions. Malar J. 2007;6:11.
12. Puett C, Coates J, Alderman H, Sadler K. Quality of care for severe acute malnutrition delivered by community health workers in southern Bangladesh. Matern Child Nutr. 2013;9(1):130-42.
13. Takasugi T, Lee AC. Why do community health workers volunteer? A qualitative study in Kenya. Public Health. 2012;126(10):839-45.
14. Karabi B, Leban K, Winch P, M. T. Community Health Worker Incentives and Disincentives: How They Affect Motivation, Retention, and Sustainability. Basic Support for Institutionalizing Child Survival Project (BASICS II) for the United States Agency for International Development; 2001. Available from: web.worldbank.org/archive/website01539/WEB/IMAGES/PNACQ722.PDF%0D. 2001.
15. Singh D, Negin J, Otim M, Orach CG, Cumming R. The effect of payment and incentives on motivation and focus of community health workers: five case studies from low- and middle-income countries. Hum Resour Health. 2015;13:58.
16. Scott K, Beckham SW, Gross M, Pariyo G, Rao KD, Cometto G, et al. What do we know about community-based health worker programs? A systematic review of existing reviews on community health workers. Hum Resour Health. 2018;16(1):39.
17. WHO. Guideline on health policy and system support to optimize community health worker programmes p. 1–116. Available from: https://apps.who.int/iris/bitstream/handle/10665/275474/9789241550369-eng.pdf?ua=1. 2018.
18. MoH. Guidelines on Continued Provision of Community Health Services in the Context of Corona Virus Pandemic in Kenya. 2020.

For peer review only

19. Wangalwa G, Cudjoe B, Wamalwa D, Machira Y, Ofware P, Ndirangu M, et al. Effectiveness of Kenya's Community Health Strategy in delivering community-based maternal and newborn health care in Busia County, Kenya: non-randomized pre-test post test study. Pan Afr Med J. 2012;13 Suppl 1:12.
20. MoH. Evaluation report of the Community Health Strategy implementation in Kenya. 2010.
21. Hensher DA, Rose JM, WH G. Applied Choice Analysis. Cambridge: Cambridge University Press; 2015.
22. Mangham LJ, Hanson K, McPake B. How to do (or not to do) ... Designing a discrete choice experiment for application in a low-income country. Health Policy Plan. 2009;24(2):151-8.
23. Blaauw D, Erasmus E, Pagaiya N, Tangcharoensathein V, Mullei K, Mudhune S, et al. Policy interventions that attract nurses to rural areas: a multicountry discrete choice experiment. Bull World Health Organ. 2010;88(5):350-6.
24. Bolduc D, Lacroix G, Muller C. The choice of medical providers in rural Benin: a comparison of discrete choice models. J Health Econ. 1996;15(4):477-98.
25. Mangham LJ, Hanson K. Employment preferences of public sector nurses in Malawi: results from a discrete choice experiment. Trop Med Int Health. 2008;13(12):1433-41.
26. Honda A, Vio F. Incentives for non-physician health professionals to work in the rural and remote areas of Mozambique--a discrete choice experiment for eliciting job preferences. Hum Resour Health. 2015;13:23.
27. Vujicic M, Alfano M, Shengelia B, Witter S. ATTRACTING DOCTORS AND MEDICAL STUDENTS TO RURAL VIETNAM: Insights from a Discrete Choice Experiment. Health, Nutrition and Population (HNP) Discussion Paper; 2010.
28. Brunie A, Chen M, Akol A. Qualitative Assessment of the Application of a Discrete Choice Experiment With Community Health Workers in Uganda: Aligning Incentives With Preferences. Glob Health Sci Pract. 2016;4(4):684-93.
29. Brunie A, Wamala-Mucheri P, Otterness C, Akol A, Chen M, Bufumbo L, et al. Keeping community health workers in Uganda motivated: key challenges, facilitators, and preferred program inputs. Glob Health Sci Pract. 2014;2(1):103-16.
30. Kenya National Bureau of Statistics MoH, National AIDS Control Council, KEMRI, NCPAD, DHS Program, ICF International. Kenya Demographic and Health Survey 2014. 2015.; 2015.
31. Helter TM, Boehler CEH. Developing attributes for discrete choice experiments in health: a systematic literature review and case study of alcohol misuse interventions. Journal of Substance Use. 2016;21(6):662-8.
32. Obadha M, Barasa E, Kazungu J, Abiiro GA, Chuma J. Attribute development and level selection for a discrete choice experiment to elicit the preferences of health care providers for capitation payment mechanism in Kenya. Health Economics Review. 2019;9(1):30.
33. Hensher DA, Rose JM, Greene WH. Applied Choice Analysis. 2 ed. Cambridge: Cambridge University Press; 2015.
34. Bridges JF, Hauber AB, Marshall D, Lloyd A, Prosser LA, Regier DA, et al. Conjoint analysis applications in health--a checklist: a report of the ISPOR Good Research Practices for Conjoint Analysis Task Force. Value Health. 2011;14(4):403-13.
35. Johnson R, Orme B. Getting the Most from CBC. Sawtooth Software, Inc.; 2003.
36. McFadden D, Train K. Mixed MNL models for discrete response. Journal of Applied Econometrics. 2000;15(5):447-70.
37. Maaya L, Meulders M, Surmont N, Vandebroek M. Effect of Environmental and Altruistic Attitudes on Willingness-to-Pay for Organic and Fair Trade Coffee in Flanders. Sustainability. 2018;10(12):4496.

For peer review only

38. Train K, Weeks M. Discrete Choice Models in Preference Space and Willingness-to-Pay Space. In: Scarpa R, Alberini A, editors. Applications of Simulation Methods in Environmental and Resource Economics. Dordrecht: Springer Netherlands; 2005. p. 1-16.
39. Hole A. Fitting mixed logit models by using maximum simulated likelihood. The Stata Journal. 2007;7(3):388-401.
40. Kok MC, Dieleman M, Taegtmeyer M, Broerse JE, Kane SS, Ormel H, et al. Which intervention design factors influence performance of community health workers in low- and middle-income countries? A systematic review. Health Policy Plan. 2015;30(9):1207-27.
41. Kok MC, Kane SS, Tulloch O, Ormel H, Theobald S, Dieleman M, et al. How does context influence performance of community health workers in low- and middle-income countries? Evidence from the literature. Health Res Policy Syst. 2015;13:13.
42. Aseyo RE, Mumma J, Scott K, Nelima D, Davis E, Baker KK, et al. Realities and experiences of community health volunteers as agents for behaviour change: evidence from an informal urban settlement in Kisumu, Kenya. Hum Resour Health. 2018;16(1):53.
43. Abdel-All M, Angell B, Jan S, Howell M, Howard K, Abimbola S, et al. What do community health workers want? Findings of a discrete choice experiment among Accredited Social Health Activists (ASHAs) in India. BMJ Glob Health. 2019;4(3):e001509.
44. Kok MC, Broerse JEW, Theobald S, Ormel H, Dieleman M, Taegtmeyer M. Performance of community health workers: situating their intermediary position within complex adaptive health systems. Hum Resour Health. 2017;15(1):59.
45. Dil Y, Strachan D, Cairncross S, Korkor AS, Hill Z. Motivations and challenges of community-based surveillance volunteers in the northern region of Ghana. J Community Health. 2012;37(6):1192-8.
46. Hensher D. Hypothetical bias, choice experiments and willingness to pay. Transportation Research. 2010;44(6):735-52.

For peer review only

BMJ Open

Incentive preferences for community health volunteers in Kenya: Findings from a Discrete Choice Experiment

Journal:	*BMJ Open*
Manuscript ID	bmjopen-2020-048059.R1
Article Type:	Original research
Date Submitted by the Author:	05-May-2021
Complete List of Authors:	Abuya, Timothy; Population Council<br>Mwanga, Daniel; Population Council Kenya, Poverty, Gender & Youth<br>Obadha, Melvin; University of Oxford, Nuffield Department of Population Health; KEMRI-Wellcome Trust Research Programme, Nuffield Department of Population Health<br>Ndwiga, Charity ; Population Council<br>Odwe, George; Population Council Kenya<br>Kavoo, Daniel; Minsitry of Health, Kenya, Division of Community Health Services<br>Wanyugu, John; Ministry of Health, Division of Community Health Services<br>Warren, Charlotte; Population Council<br>Agarwal, Smisha; Johns Hopkins Bloomberg School of Public Health, Department of International Health
**Primary Subject Heading**:	Health policy
Secondary Subject Heading:	Health services research
Keywords:	Health policy < HEALTH SERVICES ADMINISTRATION & MANAGEMENT, International health services < HEALTH SERVICES ADMINISTRATION & MANAGEMENT, Organisation of health services < HEALTH SERVICES ADMINISTRATION & MANAGEMENT, Human resource management < HEALTH SERVICES ADMINISTRATION & MANAGEMENT

SCHOLARONE™
Manuscripts

I, the Submitting Author has the right to grant and does grant on behalf of all authors of the Work (as defined in the below author licence), an exclusive licence and/or a non-exclusive licence for contributions from authors who are: i) UK Crown employees; ii) where BMJ has agreed a CC-BY licence shall apply, and/or iii) in accordance with the terms applicable for US Federal Government officers or employees acting as part of their official duties; on a worldwide, perpetual, irrevocable, royalty-free basis to BMJ Publishing Group Ltd ("BMJ") its licensees and where the relevant Journal is co-owned by BMJ to the co-owners of the Journal, to publish the Work in this journal and any other BMJ products and to exploit all rights, as set out in our licence.

The Submitting Author accepts and understands that any supply made under these terms is made by BMJ to the Submitting Author unless you are acting as an employee on behalf of your employer or a postgraduate student of an affiliated institution which is paying any applicable article publishing charge ("APC") for Open Access articles. Where the Submitting Author wishes to make the Work available on an Open Access basis (and intends to pay the relevant APC), the terms of reuse of such Open Access shall be governed by a Creative Commons licence – details of these licences and which Creative Commons licence will apply to this Work are set out in our licence referred to above.

Other than as permitted in any relevant BMJ Author's Self Archiving Policies, I confirm this Work has not been accepted for publication elsewhere, is not being considered for publication elsewhere and does not duplicate material already published. I confirm all authors consent to publication of this Work and authorise the granting of this licence.

For peer review only

Incentive preferences for community health volunteers in Kenya: Findings from a Discrete Choice Experiment

Short Title: Understanding community health volunteer incentive preferences.

Timothy Abuya[1], Daniel Mwanga[1], Melvin Obadha[2,3], Charity Ndwiga[1], George Odwe[1], Daniel Kavoo[4], John Wanyugu[4], Charlotte Warren[5] and Smisha Agarwal[6]

1. Population Council, Nairobi, Kenya

2. Health Economics Research Centre, Nuffield Department of Population Health, University of Oxford, Oxford, United Kingdom

3. Health Economics Research Unit, KEMRI-Wellcome Trust Research Programme, Nairobi, Kenya

4 Ministry of Health, Division of Community Health Services, Kenya

5. Population Council, Washington DC

6 Department of International Health, Johns Hopkins Bloomberg School of Public Health, Baltimore, Maryland, USA

Correspondence to:

Dr. Timothy Abuya

Population Council, Kenya

tabuya@popcouncil.org

For peer review only

ABSTRACT

Background

Community Health Volunteers (CHVs) play crucial roles in enabling access to health care at the community levels. Although CHVs are considered volunteers, programs provide financial and non-financial incentives. However, there is limited evidence on which bundle of financial and non-financial incentives are most effective for their improved performance.

Methods

We used a discrete choice experiment (DCE) to understand incentive preferences of CHVs with the aim to improve their motivation, performance, and retention. Relevant incentive attributes were identified through qualitative interviews with CHVs and with their supervisors. We then deployed a nominal group technique to generate and rank preferred attributes among CHVs. We developed a DCE based on the five attributes and administered it to 211 CHVs in Kilifi and Bungoma Counties in Kenya. We used mixed multinomial logit models to estimate the utility of each incentive attribute and calculated the trade-offs the CHWs were willing to make for a change in stipend.

Results

Transport was considered the incentive attribute with most relative importance followed by tools of trade then monthly stipend. CHVs preferred job incentives that offered higher monthly stipends even though it was not the most important. They had negative preference for job incentives that provided award mechanisms for the best performing CHVs as compared to jobs that provided recognition at the community level and preferred job incentives that provided more tools of trade compared to those that provided limited tools.

For peer review only

Conclusions

A bundled incentive of both financial and non-financial packages is necessary to provide a conducive working environment for CHVs. The menu of options relevant for CHVs in Kenya include transport, tools of trade and monthly stipend. Policy decisions should be contextualized to include these attributes to facilitate CHW satisfaction and performance.

Strengths and Limitation of the study

Strengths

- DCE enabled us to study incentive packages that did not exist and quantify CHVs preferences for the attributes of incentive alternatives, relative importance they placed on the attributes, and trade-offs of incentive attributes CHVs were willing to make.
- Data generated from this method provide useful information for policy makers on the attributes of a good incentive package for CHVs in Kenya and other similar contexts.
- DCE results illustrated heterogeneity illustrating the value of diversity and how that can be used to ensure context specific adaptation of the menu of incentives that a country can adopt to make use of limited resources.

Limitations

- DCE methods are prone to bias as they present hypothetical alternatives to respondents. Furthermore, our DCE adopted a forced-choice elicitation format (lack of opt-out) which might have exacerbated hypothetical bias and affected our Willingness to accept estimates.

For peer review only

INTRODUCTION

The shortage of health workers is worsening globally estimated at 18 million health workers by 2030 and 4.3 million in Africa and Asia (1-3). Recruitment and training of community health workers (CHWs) increase access to basic health services including health education where the formal sector falls short (1, 4, 5). Although CHWs roles, recruitment, remuneration and training vary (1), they work within their own community to promote health (6). CHW programs have reported substantial improvements in healthcare outcomes but struggled to maintain quality at scale (5, 7, 8). While CHW are considered volunteers (9), programs provide financial and non-financial incentives (10), facilitating their retention and improved performance (11-13). Integrating CHW into the formal health sector requires adequate incentives (14-16). However, limited evidence exist on which bundle of financial and non-financial incentives are most effective (5, 17).

In Kenya Community Health Services (CHS) are implemented through community health units (CHUs), each serving a population of 5,000 people. Community health volunteers (CHV) who serve these units are chosen by the community and trained to create demand for preventive services. CHV are supervised by a Community Health Extension Workers (CHEW) who provide training and technical support. Currently Kenya has an estimated 6,359 CHUs and 63,590 CHVs. There are 1,500 CHEWs (18). CHS in Kenya increase attendance of antenatal care visits, deliveries by skilled birth attendants, intermittent preventive treatment, testing for HIV during pregnancy, exclusive breastfeeding, and hygiene practices (19, 20). Despite this evidence of effectiveness, CHVs are considered as volunteer health workers. The government directed that they are paid a stipend of KES 2,000 per month (~20 USD), however this compensation continues to be ad-hoc mostly from non-governmental organizations (NGO) and development partners. This is often in

For peer review only

the form of transport reimbursement, lunches or a monthly stipend that is fixed or performance based. There are efforts to mainstream remuneration of CHVs with a few counties legislating county community health bills that sets aside funding for CHS.

Stated preference elicitation methods such as discrete choice experiments (DCE) can help better understanding of incentive preferences and tradeoffs CHVs are willing to make(21). Financial remuneration as well as non-financial incentives are critical to improve performance and retention of CHVs, as recommended by the 2018 WHO guideline on health policy and system support to optimize CHW programs(17). This study uses a DCE to understand incentive preferences of CHVs with the aim to improve motivation, performance, and retention of CHVs. Although DCE methods have been applied in health research in high-income countries, there are few examples of DCE used in low income setting for various cadres of health workers (22-27) and especially among CHWs (5, 28, 29). This study provides empirical evidence on the job incentive structure necessary to contribute to the welfare of CHVs using DCE.

METHODS

Phase 1: Identifying attributes and levels.

The study was conducted among CHVs from two sub counties of Kilifi, a rural coastal county, and Bungoma, a western County. Both counties reflect a range of commonly observed barriers to care, including geographical access constraints and cultural vulnerabilities (30). The Counties were purposefully selected in collaboration with County Health Management Teams to include a functional CHS where CHVs conduct routine visits, collect data, and receive some form of incentives. Additionally, the selected sites had several collaborative projects between NGOs and the government being implemented to strengthen local CHS through capacity building and improve access to health information.

For peer review only

Phase one identified financial and non-financial attributes and levels that influence CHV performance. We conducted four focus group discussions (FGDs) with CHVs and another four with their supervisors, the CHEWs. FGDs examined their understanding of incentives, preferred incentives, and barriers to implementation and the feasibility of implementing the incentives. We then deployed a nominal group technique to generate and rank preferred attributes among CHVs and CHEWs. At the national level, we conducted four in-depth interviews with policymakers at the division of community health and three key organizations that implement CHS programs.

Validation and refining attributes

Seventeen attributes and their corresponding levels were generated and subjected to a validation process by partners and policy makers at the division of community health. Key considerations used were relevance, ability to compute willingness to accept (WTA) measures, correlations between attributes, importance of an attribute, plausibility, capability of being traded and attribute non-attendance (31, 32). The process resulted in eight attributes which were further reduced to five through a pilot study (table 1).

Table 1: Attributes and attribute levels included in the DCE.

Attribute	Description	Attribute level
Recognition	Defined as any form of mechanism that help CHV be recognized at either community or facility level. This was described either as an award system or given priority in various community meetings	Recognition at community level (recognition during public meetings)
		Recognition at facility (Priority for service provision for them and their families, opportunity to work at facility as volunteer, Acknowledgment of CHV referrals and feedback)
		Award mechanism for the best performing CHVs (certification, provision of gifts, wall of fame, opportunities for exchange visit)
Income		Provision of seed money/Grant KES 100000/CHU

For peer review only

generating activities (IGA)	Some form of seed money to support income generating activities that are locally relevant	Provision of seed money/Grant KES 150000/CHU
Monthly Stipend	Financial renumeration that is payable on monthly basis	KES 2500[1]
		KES 4000
		KES 5500
Transport	Any form of support that will faicliates CHV movement from one place to another. It could be bicycles or motorcycles	Bicycles for CHV
		Motorcycles for CHV per CHU
		Motorcycle for CHEW and bicycle for CHV
Tools of trade	Availability of supplies ranging from commodities for promotive preventive activities eg drugs, job aids or IEC materials, items that identify CHV in communities or safety gears such as raincoats to support during bad weather.	Supplies and commodities + non-pharmaceutical + job aids/ Information, Education and Communication (IEC) materials
		Supplies and commodities + non-pharmaceutical + job aids/IEC materials) + Identification (badges, branded jacket/bag)
		Supplies and commodities + non-pharmaceutical + job aids/IEC materials) + Identification (badges, branded jacket/bags) + Safety gears (Raincoats, gumboots, and umbrella)

Experimental design and construction of choice tasks

Phase two comprised a cross sectional quantitative DCE survey among CHVs to elicit their preferences. The choice experiment was unlabeled consisting of two hypothetical incentive alternatives in a forced-choice elicitation format which included full profiles where all five attributes appeared in each alternative. A fractional factorial experimental design was used to generate 12 choice tasks. An orthogonal design was used where each attribute was statistically independent of each other and balanced using Sawtooth Software (33). A check for dominant

[1] 1$=Kenya shilling 100

For peer review only

alternatives was conducted and included as an extra choice task to act as a rationality test and assess internal validity of the data (34). The choice tasks were designed into a survey questionnaire. Figure 1 shows a sample of the choice set that was presented to the participants.

DCE survey questionnaire

The survey questionnaire covered background characteristics, sources of income, current incentive structure and compensation, workload, supervision, training, and the choice tasks. The Research Assistants were trained on how to explain each attribute and levels to the participants. They introduced the choice tasks by explaining to the participant the different scenarios and asked given a choice, which of the alternatives they would prefer. This was repeated for each of the choice tasks with participants selecting preferred alternative. The choice tasks were programmed to appear in a random order to minimize social desirability bias (34).

Sampling

A rule of thumb by Johnson and Orme (35) was used to determine the minimum sample size represented in the equation below:.

$$N > \frac{500c}{(t * a)}$$

Using a main effects model, a minimum sample size *N* of 84 was derived where *c* (largest number of levels *c* among the five attributes) was four, *t* was 12 choice tasks and *a* was two alternatives. To derive the sample size, CHVs were sampled from two socio-economically and culturally diverse counties namely- Kilifi and Bungoma. From each County two Sub-Counties were selected in consultation with the respective County Health Management Teams (CHMT). In Kilifi the study was conducted in Kilifi North and Kaloleni Sub-Counties while in Bungoma the study was conducted in Tongaren and Webuye West. Potential participants were recruited from all active CHWs selected with the help of the local community focal person. From each ward a maximum

For peer review only

of 13-14 active CHVs were targeted for the survey. From this process 211 CHVs were identified (109 in Kilifi, 102 in Bungoma). We administered the DCE to all CHVs after obtaining consent. All who turned up agreed to complete the DCE survey yielding 100% response rate.

Data collection

Data collection was done electronically using Open Data Kit (ODK) Programme by trained interviewers between October 14 to October 22, 2019. Before data collection, a field pre-test was conducted to practice the tool and gather experiences on the best approaches of how to explain the choice tasks. The experiences from the field pretest were used by the data collection team to refine how to explain the choice tasks to the CHVs. CHVs were presented with 12 choice tasks prompting them to choose an option they preferred among two incentive alternatives.

Data analysis

Analysis of DCE data follows random utility theory described in the published study protocol (5). A mixed multinomial logit model (MMNL) was used to estimate choice probabilities and was preferred over a conditional logit model because it fully relaxes the assumption of independence of irrelevant alternatives (IIA), considers inter respondent preference heterogeneity and within respondent correlation (33, 36). The utility function of the MMNL main effects model was specified as follows:

$$U_{njt} = \beta_0 + \beta_1 * Recognition\ at\ facility_{njt} + \beta_2 * Award\ mechanism_{njt} + \beta_3 * Income\ Generating\ Activities_{njt} + \beta_4 * Monthly\ stipend_{njt} + \beta_5 * Motorcycle_{njt} + \beta_6 * Motorcycle\ and\ Bicycle_{njt} + \beta_7 * Identification_{njt} + \beta_8 * Identification\ and\ safety\ gear_{njt} + \varepsilon_{njt}$$

Where U_{njt} was the utility a CHV n derived from selecting incentive alternative j in choice task t, β_0 was the alternative specific constant (ASC) for alternative A, β_1 to β_8 were parameters to be estimated, ε_{njt} were error terms which were assumed to be IID following type-1 extreme value distribution, monthly stipend was a continuous attribute in Kenya shillings (KES), the rest were

dummy coded variables for levels of the recognition, transport, IGA, and tools of trade attributes. In the model, all parameters of the attributes were treated as random and normally distributed except monthly stipend which was restricted to a lognormal distribution. The MMNL model resulted in means which represented choice probabilities or preferences and standard deviations which captured preference heterogeneity. The models were stratified by the two counties. Relative importance estimates were computed using the means from the panel MMNL main effects model. We took the absolute value of the mean of each attributes' parameters and multiplied it by the difference between the attribute-levels' highest and lowest values (37). This gave the maximum effect. The relative importance values were then calculated by considering the proportion of the maximum effect in the context of the total for each attribute (37).

To further explore preference heterogeneity, we explored interaction effects in the panel MMNL model by introducing interactions between incentive attributes and CHVs' characteristics; monthly stipend and sex of CHV, monthly stipend and age of CHV, IGA and sex of CHV, and IGA and age of CHV. We computed WTA estimates using a panel MMNL model in willingness to pay (WTP) space (38). We expressed the utility function in WTP space as follows

$$U_{njt} = \beta_{4n}(Monthly\ stipend_{njt} + \beta_0 + \beta_1 * Recognition\ at\ facility_{njt} + \beta_2 * Award\ mechanism_{njt} + \beta_3 * IGA_{njt} + \beta_5 * Motorcycle_{njt} + \beta_6 * Motorcycle\ and\ Bicycle_{njt} + \beta_7 * Identification_{njt} + \beta_8 * Identification\ and\ safety\ gear_{njt}) + \varepsilon_{njt}$$

The monetary attribute was monthly stipend and represented the amount of money CHVs were willing to accept per month in Kenya shillings (KES). WTA estimates were stratified by county. All models used 1000 Halton draws. Analysis was conducted using Mixlogit and Mixlogitwtp

commands on Stata 15.1(39). Chi-square test was used to compare the categories and t-test was used for continuous variables where appropriate.

Ethical Approval

The research protocol was approved by the Population Council's Institutional Review Board (PC IRB 872) and AMREF Ethical Review Board ESRC P572-2018. All the participants gave written consent for the DCE survey.

Patient and Public Involvement

There was no patient involvement.

RESULTS

Characteristics of respondents

Of 211 CHVs interviewed, the majority were female (70%), mean age was 46 years, and had secondary education (62%). More than a half (56%) reported aspiring to do business and about a third wanted to continue working as CHVs. The majority (91%) reported having been engaged in other economic activities, before becoming CHV, mainly doing small scale business and agricultural (Table 2).

Table 2: Characteristics of CHV interviewed

	Kilifi		Bungoma		Total		p-value
**Gender**	**n=109**	**%**	**n=102**	**%**	**n=211**	**%**	
Male	38	34.9	26	25.5	64	30.3	
Female	71	65.1	76	74.5	147	69.7	0.139
**Age (years) (mean (sd))***	45.5 (10.7)		46.5 (10.0)			46.0 (10.3)	0.458
**Highest Education Level**							
Primary	52	47.7	9	8.8	61	28.9	
Secondary	47	43.1	84	82.4	131	62.1	
College/University/Vocational	10	9.2	9	8.8	19	9	<0.001

For peer review only

**Future Career plans**							
Starting a business	68	62.4	49	48	117	55.5	0.036
Continue working as CHV	47	43.1	21	20.6	68	32.2	<0.001
Other options	25	22.9	28	27.5	53	25.1	0.45
Health related work	17	15.6	21	20.6	38	18	0.346
Further Study	10	9.2	6	5.9	16	7.6	0.367
Becoming a political Leader	2	1.8	1	1	3	1.4	0.6
**Marital Status**							
Married/living together	81	74.3	93	91.2	174	82.5	
Widowed	12	11	6	5.9	18	8.5	
Never married	6	5.5	2	2	8	3.8	
Divorced/separated	10	9.2	1	1	11	5.2	0.007
**Working before becoming CHV (%)**	92	84.4	99	97.1	191	90.5	0.002
**Activities engaged before becoming CHV**							
Business owner	49	53.3	41	41.4	90	47.1	
Agriculture	12	13	63	63.6	75	39.3	
Other	32	34.8	15	15.2	47	24.6	
Community/Religious Leader	5	5.4	4	4	9	4.7	<0.001
Teaching	2	2.2	5	5.1	7	3.7	
Other health professional	1	1.1	5	5.1	6	3.1	
Village Savings Group	1	1.1	0	0	1	0.5	
**Time taken to farthest household**							
Less than 15 minutes	11	10.1	4	3.9	15	7.1	
15-less than 30 minutes	44	40.4	27	26.5	71	33.6	
30-less than 60 minutes	38	34.9	52	51	90	42.7	
1 hour -less than 2 hours	15	13.8	11	10.8	26	12.3	
2 hours or more	1	0.9	8	7.8	9	4.3	0.004

**t-test was used to compare the age groups.*

Current incentive structure for CHVs in Kenya

Table 3 shows that nearly all the CHVs interviewed (98%) received some form of compensation either financial or non-financial which were often infrequent (44.2%). Among those reporting receipt of financial compensation, less than 15% received amounts that were within the recommended range of KES 2000 (US $ 20) per month while about 40% received about KES 5000 (US $ 50) per month.

For peer review only

Table 3: Type of incentives currently provided

	Kilifi		Bungoma		Total		p-value
**% receiving**	**n=109**	**%**	**n=102**	**%**	**n=211**	**%**	
Any form of compensation	107	98.2	100	98	207	98.1	0.947
**Type of compensation received**							
Financial	71	66.4	64	64	135	65.2	
Non-financial	1	0.9	0	0	1	0.5	
Both	35	32.7	36	36	71	34.3	0.565
**Frequency of receipt of compensation**							
Monthly	23	21.7	11	11	34	16.5	
Quarterly	0	0	79	79	79	38.3	
Semi-Annual	2	1.9	0	0	2	1	
Ad hoc	81	76.4	10	10	91	44.2	<0.001
**Amount received per month**							
< KES1000	86	81.1	3	3	89	43.2	
KES 1000-2499	12	11.3	17	17	29	14.1	
KES 2500 - 4999	5	4.7	1	1	6	2.9	
KES 5000 or more	3	2.8	79	79	82	39.8	<0.001
**Sources of Financial Compensation**							
Government	9	8.5	91	91	100	48.5	<0.001
NGO	99	93.4	19	19	118	57.3	<0.001
Community members	0	0.0	0	0.0	0	0.0	NA
Do not know	2	1.9	0	0	2	1	NA
**Type of Non-Financial Compensation**							
Food, clothes /other material goods	33	91.7	10	27.8	43	59.7	<0.001
Supplies for work	7	19.4	23	63.9	30	41.7	<0.001
Additional training	2	5.6	12	33.3	14	19.4	0.003
Other forms	3	8.3	2	5.6	5	6.9	0.643
Health services	0	0	1	2.8	1	1.4	NA
**Sources of Non-Financial Compensation**							
Government	10	27.8	3	8.3	13	18.1	0.032
NGO	29	80.6	31	86.1	60	83.3	0.527
Community members	1	2.8	4	11.1	5	6.9	0.164

Notes: US $ 1 = KES 100

CHV preferences and relative importance estimates

For peer review only

Table 4 provides the preference estimates of the incentives attributes. Overall, as well as independently in Kilifi and Bungoma counties, CHVs had a negative preference for jobs that provided motorcycles per CHU compared to those that provided bicycles only. Additionally, CHVs significantly preferred jobs that provided more tools of trade to those that provided limited tools. Overall, there was significant heterogeneity in preference across respondents for most attributes as denoted by the statistically significant standard deviations.

Table 4: Main effects panel MMNL model: preference estimates

		Whole sample		Kilifi		Bungoma	
**Attribute**	**Attribute level**	**β**	**SE**	**β**	**SE**	**β**	**SE**
ASC	ASC	Ref. (0)					
	ASC μ	0.138	0.192	-0.191	0.277	0.555	0.320
	ASC σ	-0.089	0.115	-0.064	0.151	-0.238	0.941
Recognition	Recognition at community level	Ref. (0)					
	Recognition at facility μ	-0.039	0.175	0.350	0.259	-0.466	0.300
	Recognition at facility σ	1.415**	0.247	1.521**	0.350	1.464**	0.456
	Award Mechanism for best performing CHV μ	-0.682**	0.186	-0.585*	0.278	-0.819**	0.277
	Award Mechanism for best performing CHV σ	1.489**	0.211	1.628**	0.299	1.532**	0.367
Transport	Bicycles for CHV	Ref. (0)					
	Motorcycle for CHV/CU μ	-2.430**	0.253	-2.309**	0.348	-2.728**	0.422
	Motorcycle for CHV/CU σ	2.098**	0.290	2.351**	0.371	1.851**	0.507
	Motorcycle for CHEW and bicycle for CHV μ	0.285	0.299	-0.506	0.423	1.337*	0.556
	Motorcycle for CHEW and bicycle for CHV σ	0.433	0.394	0.164	0.373	-0.891	1.035
Tools of trade	Supplies and commodities+ non-pharmaceutical +	Ref. (0)					

For peer review only

	job aids/IEC materials)						
	Supplies and commodities+ non-pharmaceutical + job aids/IEC materials) + Identification μ	1.330**	0.161	1.424**	0.242	1.386**	0.271
	Supplies and commodities+ non-pharmaceutical + job aids/IEC materials) + Identification σ	-0.562*	0.283	-0.060	0.140	0.924*	0.359
	Supplies and commodities+ non-pharmaceutical + job aids/IEC materials) + Identification + Safety gears μ	2.281**	0.225	2.746**	0.346	1.836**	0.306
	Supplies and commodities+ non-pharmaceutical + job aids/IEC materials) + Identification + Safety gears σ	1.149**	0.255	1.160**	0.346	1.113*	0.516
IGA	KES 100,000	Ref. (0)					
	KES 150,000 μ	-0.119	0.107	-0.109	0.170	-0.146	0.147
	KES 150,000 σ	0.114	0.106	0.184	0.333	0.141	0.106
Monthly Stipend (Thousands of KES)	Monthly stipend μ	0.543**	0.269	0.470**	0.175	0.697**	0.175
	Monthly stipend σ	0.766**	0.153	0.865	0.984	0.753**	0.236
Decision makers		211		109		102	
Observations		5,064		2,616		2,448	
Log likelihood		-1088.899		-554.010		-521.411	
McFadden's R^2		0.104		0.129		0.085	
Akaike's Information Criterion		2213.798		1144.02		1078.822	
Bayesian Information Criterion		2331.336		1249.669		1183.276	

Notes: All attributes were random and normally distributed except monthly stipend which was restricted to a lognormal distribution. Values of monthly stipend are in thousands of KES. Statistical significance: ** at 0.01 level, * at 0.05 level. β is the coefficient, SE is the robust standard error, μ is the mean, σ is the standard deviation, and ASC is alternative specific constant for alternative. All models used 1000 Halton draws

For peer review only

Relative importance estimates indicate that transport was considered the most important incentive attribute followed by tools of trade and monthly stipend. The least important incentive attribute was IGA. However, in Kilifi County, CHV considered tools of trade as the most important attribute followed by transport and monthly stipend. This was slightly different from Bungoma County who CHVs rated transport as the most important attribute followed by monthly stipend and then tools of trade.

CHVs WTA estimates

Table 5 shows WTA values in thousands of Kenya Shillings (KES). Considering WTA estimates, in the whole sample, CHVs were significantly willing to accept a KES 1060 (US $ 10.60) increase in monthly stipend for an incentive package that awarded the best performing CHV to one that provided recognition at the community level if everything else was kept constant. Additionally, CHVs were willing to accept a KES 4805 (US $ 48.05) increase in monthly stipend for an incentive package that provided motorcycles to one that provided bicycles only. The magnitude of the increase was greater among CHVs in Kilifi county KES 5917 (US $ 59.17) than Bungoma KES 3913 (US $ 39.13).

Table 5: Main effects MMNL model in WTP space

		Whole sample		Kilifi		Bungoma	
**Attribute**	**Attribute level**	**β**	**SE**	**B**	**SE**	**β**	**SE**
ASC	ASC	Ref. (0)					
	ASC μ	0.015	0.405	0.905	1.118	-0.461	0.255
	ASC σ	0.516	0.268	-0.293	2.632	-0.355	0.194
Recognition	Recognition at community level	Ref. (0)					
	Recognition at facility μ	0.025	0.398	-1.004	1.056	0.746	0.418
	Recognition at facility σ	2.821**	0.771	3.672*	1.446	-2.250**	0.358
	Award Mechanism for	1.060**	0.375	1.169	0.841	0.769*	0.328

For peer review only

	best performing CHV μ						
	Award Mechanism for best performing CHV σ	2.999**	0.636	4.261*	1.648	-1.816**	0.182
Transport	Bicycles for CHV	Ref. (0)					
	Motorcycle for CHV/CU μ	4.805**	0.819	5.917*	2.481	3.913**	0.338
	Motorcycle for CHV/CU σ	4.271**	0.972	6.047**	1.916	2.524**	0.337
	Motorcycle for CHEW and bicycle for CHV μ	-0.183	0.677	1.755	1.984	-1.317**	0.381
	Motorcycle for CHEW and bicycle for CHV σ	0.776	0.630	-0.859	1.290	1.188**	0.185
Tools of trade	Supplies and commodities+ non-pharmaceutical + job aids/IEC materials)	Ref. (0)					
	Supplies and commodities+ non-pharmaceutical + job aids/IEC materials) + Identification μ	-2.514**	0.657	-3.551**	1.341	-1.504**	0.315
	Supplies and commodities+ non-pharmaceutical + job aids/IEC materials) + Identification σ	-0.317	0.483	-0.098	0.465	-0.563*	0.284
	Supplies and commodities+ non-pharmaceutical + job aids/IEC materials) + Identification + Safety gears μ	-4.281**	1.131	-6.935**	2.428	-2.173**	0.348
	Supplies and commodities+ non-pharmaceutical + job aids/IEC materials) + Identification + Safety gears σ	2.009**	0.640	2.824	1.473	1.297**	0.209

For peer review only

IGA	KES 100,000	Ref. (0)					
	KES 150,000 μ	0.190	0.224	0.164	0.607	0.217	0.148
	KES 150,000 σ	0.159	0.353	-0.486	1.937	0.025	0.136
Monthly Stipend (Thousands of KES)	Monthly stipend μ	0.715*	0.302	0.449	0.399	2.001	1.342
	Monthly stipend σ	0.524	0.439	0.212	0.675	2.916	3.664
Decision makers		211		109		102	
Observations		5,064		2,616		2,448	
Log likelihood		-1099.937		-559.880		-525.174	
Akaike's Information Criterion		2235.874		1155.759		1086.348	
Bayesian Information Criterion		2353.412		1261.409		1190.803	

Notes: All attributes were random and normally distributed except monthly stipend which was restricted to a lognormal distribution. WTP values and monthly stipend are in thousands of KES. Statistical significance: ** at 0.01 level, * at 0.05 level. β is the coefficient, SE is the robust standard error, μ is the mean, σ is the standard deviation, and ASC is alternative specific constant for alternative. All models used 1000 Halton draws

Furthermore, CHVs in the whole sample were willing to accept a KES 2514 (US $ 25.14) reduction in monthly stipend for an incentive package that additionally provided identification and a KES 4281 (US $ 42.81) decrease for an incentive package that additionally provided identification and safety gears to one that only provided supplies and commodities, non-pharmaceuticals, and job aids when everything else was kept constant. These reductions in monthly stipends were also greater in Kilifi County than Bungoma. Overall, there was significant heterogeneity in WTA estimates across respondents in most attributes as denoted by the statistically significant standard deviations.

Interactions

Table 6 shows the model that present results of the interactions between incentive attributes and CHVs' characteristics. Results show that younger CHVs significantly preferred higher salaries when everything else was kept constant. The interaction between monthly stipend and gender suggested that male CHVs preferred higher salaries than their female counterparts if everything

For peer review only

else is kept constant. However, this interaction was not statistically significant. Overall, both interactions revealed significant heterogeneity in preferences across respondents.

Table 6: MMNL Model with Interactions (Gender and Age of the CHV): Preference estimates

**Attribute**	**Attribute level**	**β**	**SE**
ASC	ASC	Ref. (0)	
	ASC μ	0.127	0.215
	ASC σ	-0.076	0.239
Recognition	Recognition at community level	Ref. (0)	
	Recognition at facility μ	-0.149	0.202
	Recognition at facility σ	1.675**	0.296
	Award Mechanism for best performing CHV μ	-0.781**	0.216
	Award Mechanism for best performing CHV σ	1.696**	0.267
Transport	Bicycles for CHV	Ref. (0)	
	Motorcycle for CHV/CU μ	-2.618**	0.331
	Motorcycle for CHV/CU σ	2.334**	0.347
	Motorcycle for CHEW and bicycle for CHV μ	0.395	0.348
	Motorcycle for CHEW and bicycle for CHV σ	0.817**	0.311
Tools of trade	Supplies and commodities+ non-pharmaceutical + job aids/IEC materials)	Ref. (0)	
	Supplies and commodities+ non-pharmaceutical + job aids/IEC materials) + Identification μ	1.536**	0.216
	Supplies and commodities+ non-pharmaceutical + job aids/IEC materials) + Identification σ	-0.653*	0.323
	Supplies and commodities+ non-pharmaceutical + job aids/IEC materials) + Identification + Safety gears μ	2.525**	0.306
	Supplies and commodities+ non-pharmaceutical + job aids/IEC materials) + Identification + Safety gears σ	1.206**	0.271
IGA	KES 100,000	Ref. (0)	
	KES 150,000 μ	-0.592	0.587
	KES 150,000 σ	0.090	0.322
Monthly Stipend (Thousands of KES)	Monthly stipend μ	1.182**	0.290
	Monthly stipend σ	0.561**	0.109
Monthly stipend ##Female	Monthly stipend ##Female μ	-0.214	0.121
	Monthly stipend ##Female σ	0.212*	0.086
Monthly stipend ##Age	Monthly stipend ##Age μ	-0.011*	0.005
	Monthly stipend ##Age σ	0.006*	0.003
IGA ##Female	IGA (KES 150,000) ##Female μ	0.133	0.224
	IGA (KES 150,000) ##Female σ	-0.085	0.154
IGA ##Age	IGA (KES 150,000) ##Age μ	0.009	0.012
	IGA (KES 150,000) ##Age σ	0.001	0.018
Decision makers		211	

For peer review only

Observations	5,064
Log likelihood	-1081.439
McFadden's R^2	0.107
Akaike's Information Criterion	2214.877
Bayesian Information Criterion	2384.655

Notes: ## denotes interactions, age is in years, monthly stipend is in thousands of KES. All attributes were random and normally distributed except monthly stipend and its interactions with gender and with age which were restricted to a lognormal distribution. Statistical significance: ** at 0.01 level, * at 0.05 level. β is the coefficient, SE is the robust standard error, μ is the mean, σ is the standard deviation, and ASC is alternative specific constant for alternative. All models used 1000 Halton draws

Discussion

Our study shows that transport was considered the most important attribute, followed by tools of trade and monthly stipend. Some variations in these preferences were observed between Kilifi and Bungoma counties perhaps due to rurality and varying social-economic contexts. Critical variations in preferences were observed based on CHV's age and gender. Our result shows that older CHVs significantly preferred incentives with lower salaries when everything else was kept constant. Perhaps because older CHV may have experienced voluntariness for long and are therefore content with lower salaries. Younger male CHVs had a stronger preference for higher salaries, compared to older female CHVs, an observation associated with gender roles, perceptions of workload, resources, and logistics, which are barriers to CHW performance (40). It is plausible that gender roles affect salary preference and may have implications on retention, a policy issue that any incentive structure need to consider (41). Workload on the other hand was discussed in the context of use of paper tools or CHWs covering large areas with expectations of reporting household visits monthly regardless of spread, limiting their ability to engage in personal activities and responsibilities (42). These result contrasts with studies from India which showed that age did not affect CHW salary preferences(43). Overall, heterogeneity in preferences across respondents is a feature that has been reported elsewhere with preferences being influenced by education level, having another paid job or being the main household earner(43).

For peer review only

CHVs had a negative preference for incentive that provided recognition at the facility and award mechanisms for the best performing CHVs as compared to jobs that provided recognition at the community level. These results are augmented by WTA estimates, which suggest that CHVs were willing to accept a KES 1060 (US $ 10.60) increase in monthly stipend for a job that awarded the best performing CHV to one that provided recognition at the community level if everything else was kept constant. Recognition was discussed as a key motivator that will enhance their work. For example, recognition at community level could be as simple as being given a chance to speak at the community public events or benchmarking to other sites to share learnings. Since CHWs are often believed to be vehicles for facilitating community agency and triggering social change(44), recognition is central to maintaining trust between communities and CHW. Community recognition has been shown to influence their status in their locality which might confer other benefits beyond their role as CHVs as has been reported in Uganda with an enhanced status being conferred to them that derive pride and greater access to help or information and being consulted on a range of problems(29). However, we observed variations in role of recognition with geographical location. For example, Kilifi County, a coastal mixed urban and rural settings unlike Bungoma an agrarian setting preferring jobs that provided recognition at the facility to recognition at community. These differences might be due to cultural orientation or benefits accrued from serving in the facility that might elevate CHVs to a same status as health facility-based providers.

Lack of transport for CHWs is reported as a factor limiting their work performance (29, 40). Our results show that CHVs had a negative preference for jobs that provided motorcycles to be used by the CHU and positive preference for jobs that provided motorcycles to the CHU and bicycles

For peer review only - http://bmjopen.bmj.com/site/about/guidelines.xhtml

to the CHV as compared to those that provided bicycles only. These results illustrate the value of transport that facilitates CHV to conduct routine visits. Preferences for bicycles for themselves and a motorcycle per CHU is probably based on the ability to improve community-facility linkages by transportation of clients to the hospital. Community referrals to a facility is a major barrier to optimal service use where clients who do not have means of transport fail to comply with referrals leading to poor outcomes. Motorcycles can also be used communally as a source of income when not busy serving patients. However, issues of maintenance cost and managing communally owned motorbikes may be an avenue for disagreement and might be a complex policy option to implement. These results echo experiences from Uganda which showed the CHW prefer programs with transport in form of bicycles, which would facilitate visiting clients or going to the health center for supervisory meetings or access supplies but if nonexistent conferred additional financial cost(29).

CHVs significantly preferred jobs that provided more tools of trade to those that provided limited tools with significant heterogeneity in preference across respondents. Having reporting tools and job aids replenished timely facilitate accuracy in reporting as well, orientation to new updates and facilitate CHW performance. Provision of identification materials such as T-shirt, Badges, Caps, branded bags, reflector jackets were described as useful especially during the rainy seasons to maintain the training materials. In addition, provision of essential requirements in the form of CHVs Kit including pharmaceuticals and non-pharmaceuticals for work were considered important. This may mean that policies that provide CHV with essential tools of trade is likely to influence their performance but will have to be structured in a way that ensures limited stock outs to facilitate continuity of service to communities. In summary other studies have shown that

For peer review only

transport constraints and lack of supplies hinder CHW performance(29, 45), thus a useful non-financial incentive.

Finally, relative importance estimates indicate that transport was considered the most important attribute followed by tools of trade and monthly stipend. The least important attribute was IGA. These findings have significant policy direction in the sense that an incentive structure which combines effective transport system for CHV, essential tools of trade to enable them to perform their roles and monthly stipend will be critical for the success of CHV program. However, the levels may need to be contextualized to ensure that the implementation is feasible and sustainable. It might also be important to consider geographical variations even within a country. For example, relative importance varied with Kilifi County considered tools of trade as the most important attribute followed by transport and monthly stipend while Bungoma CHVs rated transport as the most important attribute followed by monthly stipend and then tools of trade. These findings support the growing evidence of the importance of the non-financial interventions in motivating CHWs in developing countries(43).

DCE is a stated preference approach that enabled us to study incentive packages that did not exist. We were able to quantify CHVs preferences for the attributes of incentive alternatives, relative importance they placed on the attributes, and quantify trade-offs of incentive attributes CHVs were willing to make. This provides useful information for policy makers on the attributes of a good incentive package for CHVs in Kenya and other similar contexts. However, since DCE is a stated preference approach, they are prone to bias as they present hypothetical alternatives to respondents. Furthermore, our DCE adopted a forced-choice elicitation format (lack of opt-out) which might have exacerbated hypothetical bias and affected our WTA estimates(46). Nonetheless, we used

For peer review only

qualitative methods to identify and validate our attributes and levels with potential study participants and policy makers which might have reduced hypothetical bias. A potential value of the DCE beyond the menu of incentives options that CHW prefer may be to practically test the results by implementing the preferred incentive attributes and assessing how it influences the motivation and their retention. Although our results show heterogeneity in terms of incentive preferences for CHVs in different locations, the strength is that it illustrates the value of diverse settings and how that can be used to ensure context specific adaptation of the menu of incentives that a country can adopt to make use of limited resources.

Conclusion

Our study confirms that a bundled incentive of both financial and non-financial packages is necessary to provide a conducive working environment for CHVs. The menu of options relevant for CHVs in Kenya include transport that was considered the most important attribute followed by tools of trade and monthly stipend. The least important attribute was IGA. Policy decisions should be contextualized to include these attributes to facilitate CHW satisfaction and performance.

For peer review only

Authorship contributions

TA co-conceptualized the study, led the analysis, and drafted the manuscript. DM Supported in data collection, overall study implementation, data management, analysis and reviewed the manuscript. MO supported in technical design of the DCE experiment, guided analysis and reviewed the manuscripts for technical content. CN supported in design of the DCE experiment and reviewed manuscript. GO supported in technical design of the DCE experiment and reviewed the manuscripts for technical content. DK and JW supported design of the DCE experiment and reviewed the manuscripts for technical content. CW and SA are lead investigators in the overall study, co-designed the DCE study, framing of this manuscript, and contributed significantly to writing and reviewing drafts. All contributing authors provided inputs and approved the final draft for submission.

Competing interests

Authors have no competing interests.

Funding

This paper is based on research funded by the Bill & Melinda Gates Foundation- ID OPP1174594. The findings and conclusions contained within are those of the authors and do not necessarily reflect positions or policies of the Bill & Melinda Gates Foundation

Data sharing statement

All the data contributing to this manuscript have been archived in accordance with Population Council data sharing policies. Data will be made available upon reasonable request from the lead Author.

For peer review only

Reference

1. O'Donovan J, O'Donovan C, Kuhn I, Sachs SE, Winters N. Ongoing training of community health workers in low-income andmiddle-income countries: a systematic scoping review of the literature. BMJ Open. 2018;8(4):e021467.
2. Darzi A, Evans T. The global shortage of health workers-an opportunity to transform care. Lancet. 2016;388(10060):2576-7.
3. Global strategy on human resources for health: Workforce 2030 [press release]. 2016.
4. Perry HB, Zulliger R, Rogers MM. Community health workers in low-, middle-, and high-income countries: an overview of their history, recent evolution, and current effectiveness. Annu Rev Public Health. 2014;35:399-421.
5. Agarwal S, Anaba U, Abuya T, Kintu R, Casseus A, Hossain S, et al. Understanding incentive preferences of community health workers using discrete choice experiments: a multicountry protocol for Kenya, Uganda, Bangladesh and Haiti. BMJ Open. 2019;9(12):e033601.
6. Turinawe EB, Rwemisisi JT, Musinguzi LK, de Groot M, Muhangi D, de Vries DH, et al. Selection and performance of village health teams (VHTs) in Uganda: lessons from the natural helper model of health promotion. Hum Resour Health. 2015;13:73.
7. Bang AT, Bang RA, Baitule SB, Reddy MH, Deshmukh MD. Effect of home-based neonatal care and management of sepsis on neonatal mortality: field trial in rural India. Lancet. 1999;354(9194):1955-61.
8. Chowdhury AM, Chowdhury S, Islam MN, Islam A, Vaughan JP. Control of tuberculosis by community health workers in Bangladesh. Lancet. 1997;350(9072):169-72.
9. Maes K. Examining health-care volunteerism in a food- and financially-insecure world. Bull World Health Organ. 2010;88(11):867-9.
10. Hongoro C, McPake B. How to bridge the gap in human resources for health. Lancet. 2004;364(9443):1451-6.
11. Nsabagasani X, Jesca Nsungwa S, Kallander K, Peterson S, Pariyo G, Tomson G. Home-based management of fever in rural Uganda: community perceptions and provider opinions. Malar J. 2007;6:11.
12. Puett C, Coates J, Alderman H, Sadler K. Quality of care for severe acute malnutrition delivered by community health workers in southern Bangladesh. Matern Child Nutr. 2013;9(1):130-42.
13. Takasugi T, Lee AC. Why do community health workers volunteer? A qualitative study in Kenya. Public Health. 2012;126(10):839-45.
14. Karabi B, Leban K, Winch P, M. T. Community Health Worker Incentives and Disincentives: How They Affect Motivation, Retention, and Sustainability. Basic Support for Institutionalizing Child Survival Project (BASICS II) for the United States Agency for International Development; 2001. Available from: web.worldbank.org/archive/website01539/WEB/IMAGES/PNACQ722.PDF%0D. 2001.
15. Singh D, Negin J, Otim M, Orach CG, Cumming R. The effect of payment and incentives on motivation and focus of community health workers: five case studies from low- and middle-income countries. Hum Resour Health. 2015;13:58.
16. Scott K, Beckham SW, Gross M, Pariyo G, Rao KD, Cometto G, et al. What do we know about community-based health worker programs? A systematic review of existing reviews on community health workers. Hum Resour Health. 2018;16(1):39.
17. WHO. Guideline on health policy and system support to optimize community health worker programmes p. 1–116. Available from: https://apps.who.int/iris/bitstream/handle/10665/275474/9789241550369-eng.pdf?ua=1. 2018.
18. MoH. Guidelines on Continued Provision of Community Health Services in the Context of Corona Virus Pandemic in Kenya. 2020.

19. Wangalwa G, Cudjoe B, Wamalwa D, Machira Y, Ofware P, Ndirangu M, et al. Effectiveness of Kenya's Community Health Strategy in delivering community-based maternal and newborn health care in Busia County, Kenya: non-randomized pre-test post test study. Pan Afr Med J. 2012;13 Suppl 1:12.
20. MoH. Evaluation report of the Community Health Strategy implementation in Kenya. 2010.
21. Hensher DA, Rose JM, WH G. Applied Choice Analysis. Cambridge: Cambridge University Press; 2015.
22. Mangham LJ, Hanson K, McPake B. How to do (or not to do) ... Designing a discrete choice experiment for application in a low-income country. Health Policy Plan. 2009;24(2):151-8.
23. Blaauw D, Erasmus E, Pagaiya N, Tangcharoensathein V, Mullei K, Mudhune S, et al. Policy interventions that attract nurses to rural areas: a multicountry discrete choice experiment. Bull World Health Organ. 2010;88(5):350-6.
24. Bolduc D, Lacroix G, Muller C. The choice of medical providers in rural Benin: a comparison of discrete choice models. J Health Econ. 1996;15(4):477-98.
25. Mangham LJ, Hanson K. Employment preferences of public sector nurses in Malawi: results from a discrete choice experiment. Trop Med Int Health. 2008;13(12):1433-41.
26. Honda A, Vio F. Incentives for non-physician health professionals to work in the rural and remote areas of Mozambique--a discrete choice experiment for eliciting job preferences. Hum Resour Health. 2015;13:23.
27. Vujicic M, Alfano M, Shengelia B, Witter S. ATTRACTING DOCTORS AND MEDICAL STUDENTS TO RURAL VIETNAM: Insights from a Discrete Choice Experiment. Health, Nutrition and Population (HNP) Discussion Paper; 2010.
28. Brunie A, Chen M, Akol A. Qualitative Assessment of the Application of a Discrete Choice Experiment With Community Health Workers in Uganda: Aligning Incentives With Preferences. Glob Health Sci Pract. 2016;4(4):684-93.
29. Brunie A, Wamala-Mucheri P, Otterness C, Akol A, Chen M, Bufumbo L, et al. Keeping community health workers in Uganda motivated: key challenges, facilitators, and preferred program inputs. Glob Health Sci Pract. 2014;2(1):103-16.
30. Kenya National Bureau of Statistics MoH, National AIDS Control Council, KEMRI, NCPAD, DHS Program, ICF International. Kenya Demographic and Health Survey 2014. 2015.; 2015.
31. Helter TM, Boehler CEH. Developing attributes for discrete choice experiments in health: a systematic literature review and case study of alcohol misuse interventions. Journal of Substance Use. 2016;21(6):662-8.
32. Obadha M, Barasa E, Kazungu J, Abiiro GA, Chuma J. Attribute development and level selection for a discrete choice experiment to elicit the preferences of health care providers for capitation payment mechanism in Kenya. Health Economics Review. 2019;9(1):30.
33. Hensher DA, Rose JM, Greene WH. Applied Choice Analysis. 2 ed. Cambridge: Cambridge University Press; 2015.
34. Bridges JF, Hauber AB, Marshall D, Lloyd A, Prosser LA, Regier DA, et al. Conjoint analysis applications in health--a checklist: a report of the ISPOR Good Research Practices for Conjoint Analysis Task Force. Value Health. 2011;14(4):403-13.
35. Johnson R, Orme B. Getting the Most from CBC. Sawtooth Software, Inc.; 2003.
36. McFadden D, Train K. Mixed MNL models for discrete response. Journal of Applied Econometrics. 2000;15(5):447-70.
37. Maaya L, Meulders M, Surmont N, Vandebroek M. Effect of Environmental and Altruistic Attitudes on Willingness-to-Pay for Organic and Fair Trade Coffee in Flanders. Sustainability. 2018;10(12):4496.

For peer review only

38. Train K, Weeks M. Discrete Choice Models in Preference Space and Willingness-to-Pay Space. In: Scarpa R, Alberini A, editors. Applications of Simulation Methods in Environmental and Resource Economics. Dordrecht: Springer Netherlands; 2005. p. 1-16.
39. Hole A. Fitting mixed logit models by using maximum simulated likelihood. The Stata Journal. 2007;7(3):388-401.
40. Kok MC, Dieleman M, Taegtmeyer M, Broerse JE, Kane SS, Ormel H, et al. Which intervention design factors influence performance of community health workers in low- and middle-income countries? A systematic review. Health Policy Plan. 2015;30(9):1207-27.
41. Kok MC, Kane SS, Tulloch O, Ormel H, Theobald S, Dieleman M, et al. How does context influence performance of community health workers in low- and middle-income countries? Evidence from the literature. Health Res Policy Syst. 2015;13:13.
42. Aseyo RE, Mumma J, Scott K, Nelima D, Davis E, Baker KK, et al. Realities and experiences of community health volunteers as agents for behaviour change: evidence from an informal urban settlement in Kisumu, Kenya. Hum Resour Health. 2018;16(1):53.
43. Abdel-All M, Angell B, Jan S, Howell M, Howard K, Abimbola S, et al. What do community health workers want? Findings of a discrete choice experiment among Accredited Social Health Activists (ASHAs) in India. BMJ Glob Health. 2019;4(3):e001509.
44. Kok MC, Broerse JEW, Theobald S, Ormel H, Dieleman M, Taegtmeyer M. Performance of community health workers: situating their intermediary position within complex adaptive health systems. Hum Resour Health. 2017;15(1):59.
45. Dil Y, Strachan D, Cairncross S, Korkor AS, Hill Z. Motivations and challenges of community-based surveillance volunteers in the northern region of Ghana. J Community Health. 2012;37(6):1192-8.
46. Hensher D. Hypothetical bias, choice experiments and willingness to pay. Transportation Research. 2010;44(6):735-52.

For peer review only

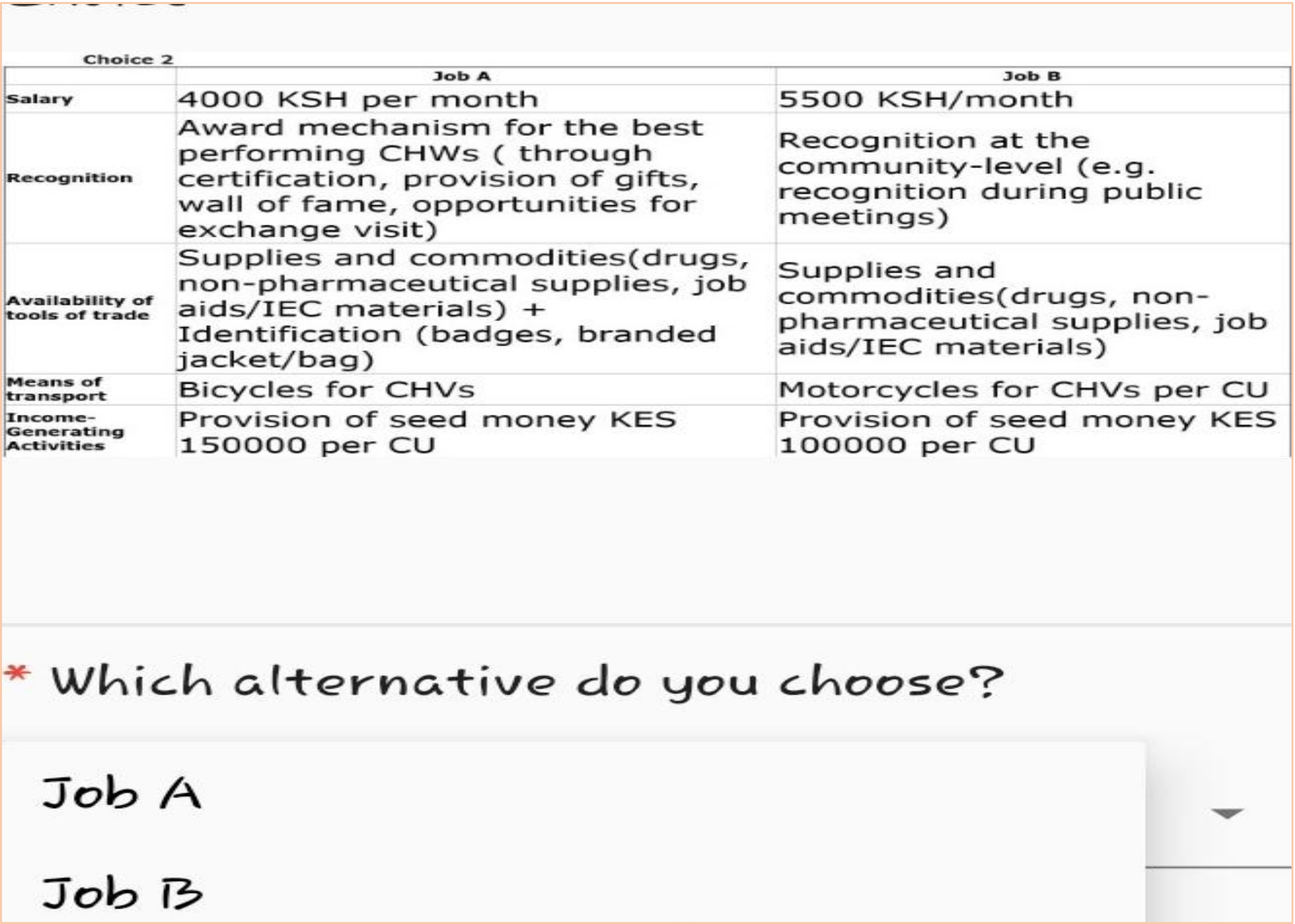

Choice 2

	Job A	Job B
Salary	4000 KSH per month	5500 KSH/month
Recognition	Award mechanism for the best performing CHWs (through certification, provision of gifts, wall of fame, opportunities for exchange visit)	Recognition at the community-level (e.g. recognition during public meetings)
Availability of tools of trade	Supplies and commodities(drugs, non-pharmaceutical supplies, job aids/IEC materials) + Identification (badges, branded jacket/bag)	Supplies and commodities(drugs, non-pharmaceutical supplies, job aids/IEC materials)
Means of transport	Bicycles for CHVs	Motorcycles for CHVs per CU
Income-Generating Activities	Provision of seed money KES 150000 per CU	Provision of seed money KES 100000 per CU

Figure 1: sample of Choice set

iew only
